# Promoting Chinese medical equipment enterprises’ environmentally friendly production through digital transformation and net zero strategic consensus

**DOI:** 10.1038/s41598-025-26285-6

**Published:** 2025-11-27

**Authors:** Yaowen Xu, Anning Zhou, Xiaozhen Wang, Qin Zeng, Setyawan Widyarto

**Affiliations:** 1 Dazhou Central Hospital, Dazhou, China; 2 Centre for Graduate Studies, Faculty of Communication Visual Arts & Computing, Universiti Selangor, Selangor, Malaysia; 3 School of business administration, Chongqing Technology and Business University, Chongqing, China; 4 Sichuan Provincial People’s Hospital, Chengdu, China

**Keywords:** Digital transformation, Net-zero strategic consensus, Environmentally friendly production, Sustainability orientation, Resource-based view, Environmental sciences, Environmental social sciences

## Abstract

The rapid growth of China’s medical industry has raised concerns about environmental sustainability, particularly regarding waste pollution from medical product production. This has led to calls for environmentally friendly production (EFP) practices in the sector. Drawing on the Resource-Based View (RBV) and Dynamic Managerial Capabilities (DMC) perspectives, this study explores the effects of digital transformation (DT) on EFP, with survey data from 316 medical equipment manufacturing companies in China. The findings depict a positive relationship between DT and EFP, mediated by net-zero strategic consensus (NZSC). Furthermore, sustainability orientation (SO) positively moderates the linkage between DT and NZSC. This study developed and validated a series of novel measurement indicators for NZSC and SO. Specifically, we measure NZSC from three dimensions: long term consensus, short term consensus, and strategic optimality; Measuring SO through knowledge, practice, and commitment to sustainability. Therefore, this study contributes to the existing literature, expanding the theoretical application of DT and RBV in the net-zero production field, and providing managerial implications for policymakers promoting EFP practices.

**Supplementary Information:**

The online version contains supplementary material available at 10.1038/s41598-025-26285-6.

## Introduction

Globally, the rapid growth of China’s medical industry, while contributing significantly to economic development, has also raised concerns about its environmental impact, particularly waste pollution from medical product production. This challenge has fueled a growing call for the adoption of environmentally friendly production (EFP) practices. Although extant works have shown that board characteristics^1^, green values^2^, pro-environmental education^3^ have a positive impact on environmentally friendly production. These studies seldom take into account the current digital transformation (i.e., the application of digital technologies such as big data, cloud computing, mobile and social media platforms, in transforming the general operation of an organization to create value) concepts, which is altering global issues^4^. Admittedly, Organizations that incorporate digital transformation (DT) into their strategic activities enhance their core business operations, ultimately creating greater value^4^. Extant works have established a significant role of DT in fostering net-zero strategic attitudes, pro-environment production^5^, and human production methods^6^.

However, there is a dearth of exploratory research on the influencing factors and internal mechanisms of the linkage between DT and emerging market medical enterprises’ environment friendly production. Therefore, this study aims to explore the effect of DT on environment friendly production (EFP). The relationship mechanisms of net-zero strategic consensuses (NZSC) and sustainability orientation on the linkage between DT and EFP have been rare. The current study contends that DT and sustainability orientation as concepts can help Chinese medical enterprises reach a NZSC and achieve EFP. Chinese market environment offers a fecund ground to understand the relationship among the variables towards cleaner production to address market and environmental needs.

Firstly, this study clarified the logical relationship between DT and EFP. Recent study indicate that DT is becoming a critical driver of enterprise transformation, enabling firms to reshape their business models and strategic planning^7^. As an indispensable tool for promoting green development, DT has been widely discussed in academia regarding its impact on green innovation^8^, corporate environmental performance^9^, and green technology innovation^10^. However, there is very little evidence of the impact of DT on EFP research. This study provides empirical evidence from Chinese medical equipment suppliers, clarifying the impact relationship between DT and EFP. And contributes to filling the current research gap.

Secondly, drawing on the perspectives of Dynamic Managerial Capabilities (DMC) and Resource-Based View (RBV) perspective, this study posits that NZSC mediates the relationship between DT and EFP. Unlike broader frameworks such as strategic alignment or green culture, NZSC specifically captures the degree of agreement among organizational members regarding the firm’s net-zero strategy and goals. It reflects a shared cognitive commitment to emission-reduction targets and pathways, an aspect not fully addressed within existing resource- or capability-based frameworks. As more companies adopt low-carbon goals, and achieving strategic consensus around net- zero objectives has become critical for their realization^11^. Given the positive role of knowledge and information resources in reaching strategic consensus, the knowledge agglomeration effects generated by DT may enhance EFP by promoting NZSC. From a DMC perspective, this study helps to open the “black box” of how DT translates into EFP, offering an important extension to existing research.

Finally, this study argues that sustainability orientation (SO) enhances the effect of DT on NZSC. SO refers to a responsible attitude and inclination towards ecology and society. Research has shown that SO can enhance sustainable business orientation^12^, promote green product innovation^13^, and effectively improve the green innovation performance of enterprises^14^. From the RBV, this study contends that SO can help and guide the knowledge resource aggregation effect of DT towards the net-zero strategic direction, thereby expanding the impact of DT on NZSC. However, the studys that analyze how SO affects the correlation between DT and NZSC in the extant literature are still deficient.

To fill these gaps, the current research draws on RBV and DMC theories to construct a theoretical framework. From the RBV perspective, this study views digital technologies, NZSC among the management team, and a strong sustainability orientation as critical resources. By effectively leveraging these resources, medical equipment manufacturers can enhance their EFP capabilities, leading to improved environmental performance and a potential competitive advantage; The DMC perspective highlights the importance of dynamic managerial capabilities in adapting to digital transformation and aligning the organization toward a common goal of net-zero production. These capabilities are essential for effectively integrating EFP principles into the organization’s operations and responding to the evolving demands of environmentally conscious markets.

To sum up, based on the RBV and DMC, this study explores the possible correlation between DT and EFP, the mediating role of NZSC, and the regulatory effect of SO. This study magnifies the theoretical application of DT, RBV, and DMC in net-zero production, which helps provide policymaking references for relevant academic inquiries and policy formulation.

## Theory and hypotheses

Digital transformation represents a fundamental shift in business models, exerting significant influence on institution, stakeholders, resources, and managerial capabilities. However, for the purpose of this study, the resource-based and capability-oriented perspective provide a more suitable theoretical framework than institutional and stakeholder theories, as they place greater emphasis on organizational behavior and outcomes.

### Resource-based view (RBV)

The Resource-Based View (RBV) offers a fundamental framework for understanding how firms achieve and sustain competitive advantage by leveraging valuable, rare, inimitable, and non-substitutable resources^15,16^. It explains firm-level performance heterogeneity as a result of differences in resource endowments, both tangible (e.g., digital infrastructure) and intangible (e.g., strategic orientation and internal consensus)^5,17,18^.

In this study, we conceptualize digital transformation (DT), sustainability orientation (SO), and net-zero strategy consensus (NZSC) as key organizational resources. When strategically configured and integrated, these resources can facilitate environmentally friendly production (EFP). However, consistent with RBV, resource possession alone is not sufficient, firms create value by effectively bundling and deploying these resources^19^. We further argue that this integration requires a well-developed transformation network^5^, robust digital infrastructure^3^, strong sustainability orientation^20,21^, and capable top management^22^.

Empirical studies have demonstrated that digital technologies^3,8^ and sustainability orientation^20,21^, significantly enhance innovation and environmental performance. Therefore, RBV provides a suitable theoretical foundation for examining how firms can leverage DT, SO, and NZSC to support EFP. This aligns with prior literature recognizing digitalization as a source of competitive advantage (e.g.,Butt^23^;Florek-Paszkowska, et al.^24^; Rashid, et al.^25^). Accordingly, this study posits that DT can positively influence environmentally friendly practices in the medical supplies sector. Moreover, by integrating DT, SO, and NZSC, we aim to clarify the mechanisms through which these resources interact to create value. In particular, SO may guide DT toward collecting and applying knowledge in an environmentally friendly direction.

### Dynamic managerial capabilities (DMC)

**Fig. 1:**
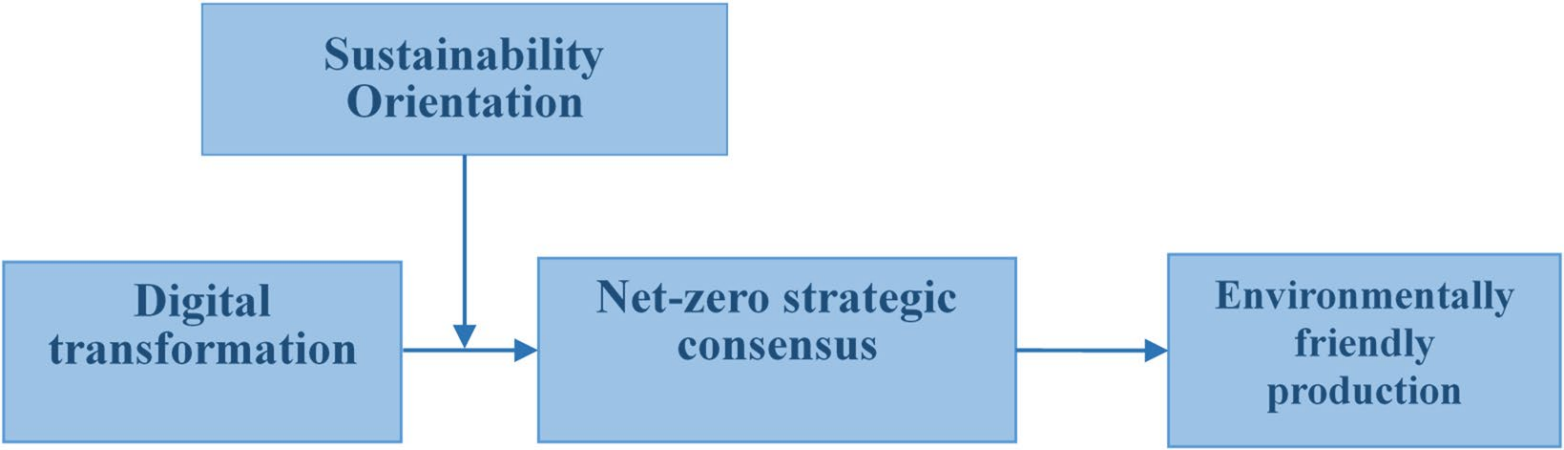
Research framework.

While RBV focuses on what resources firms have, the DMC perspective focuses on how managerial actions shape the deployment and reconfiguration of those resources in dynamic environments^26^. The concept contends that enterprises should continuously align, modify, and reconfigure their resources and capabilities in unstable environments to safeguard sustained innovation^27^. It exemplifies the significance of managerial intent, routines, and agility in swaying the reformation of an enterprise’s resource base^28^ to improve its efficiency and effectiveness toward a sustainable competitive advantage^26,27^. Hence, the DMC perspective is well-suited for investigating environmental transformation^29^, as it offers theoretical clarity on how managerial actions drive the adaptation and reconfiguration of resources, processes, and structures essential for implementing EFP through effective DT^30^. Based on this, we contend that enterprises NZSC play a significant function in promoting the implementation of EFP via DT (which focuses on the collection of resources related to environmental protection). To this end, this study scrutinizes the intrinsic mechanism of DT on EFP, applying the perspectives of RBV and DMC. First, we examine the relationship between DT and EFP. Second, we develop moderating (SO) and mediating (NZSC) concepts linking SO and NZSC on the relationship between DT and EFP. In summary, we have established a research framework based on the RBV and DMC on DT, NZSC, EFP, and SO, as shown in Fig. 1.

### The linkage amongst DT and EFP

DT refers to the integration of digital technology into all aspects and operations of the organization and the adoption of disruptive technologies to improve productivity, value creation, and social welfare^31–33^. This concept facilitates the strategic use of digital technologies to enhance operational efficiency, enrich customer engagement, simplify internal processes, and enable the creation of novel business models^34,35^. Referring to Almaqtari, et al.^1^, this paper defines EFP as a production activity that considers environmental factors in product design, material selection, production process, consumer payment, and scrap management. In recent years, green development has emerged as a pressing priority for many economies, especially in developing countries, as they strive to establish effective governance frameworks for environmental sustainability^36^. Drawing on the RBV, enterprises adopting digital technologies can enhance green activities to create value^8^. Hence, the present study contends that DT can improve green development issues (e.g., EFP activities) to address environmental and customer demands. From the RBV, in the process of DT, through the popularization and expanded application, big data, cloud computing, artificial intelligence, mobile and social media, and other digital technologies^37^ can promote the in-depth development of EFP. Predominantly manifested in two facets: first, DT can promote the sharing, integration, and development of relevant knowledge in the field of EFP to effectively solve problems such as information departmentalization, fragmentation, and information asymmetry and form a complete data information system. For instance, under the influence of knowledge exchange on pro-environmental green development, the values and decision-making concepts of corporate executives will lean towards ecological friendliness, thereby promoting the internal implementation of EFP within the enterprise; On the other hand, DT can promote knowledge integration in heterogeneous industries, break information silos, and grasp market development trends and consumer demands. Thus, it promotes the enterprises’ ecological and sustainable development by acquiring and absorbing advanced green development concepts, models, and green production technologies from external sources. From the existing literature, scholars attest that there is a significant association between DT and green innovation^8^, corporate environmental performance^9^, and green technology innovation^10^.

In the context of the Chinese market environment, we argue that DT can promote product technology upgrading and business process upgrading in the environmental field, thereby effectively promoting resource sharing and resource optimization, meeting the data element requirements of green development, and ultimately enhancing or optimizing EFP levels^10^ to create EFP. Based on this, we state that:

#### H1

 DT has a positive effect on EFP.

### The mediating effect of NZSC

Grounded in the RBV, we argue that DT facilitates the acquisition, integration, and reconfiguration of environmental information resources, enabling firms to build capabilities that support their net-zero goals and ultimately enhance EFP. In recent years, an increasing number of companies have committed to net-zero targets and incorporated science-based emission reduction milestones into their strategic roadmaps^11^. However, the effectiveness of such strategic goals depends not only on technological capabilities, but also on the internal alignment and collective commitment of organizational members.

To capture this internal alignment, we define NZSC as the degree to which members agree on the company’s net-zero strategy and related goals. This definition draws upon the strategic consensus literature, which emphasizes agreement among organizational members on long-term, short-term, and survival-related strategies^38^, and adapts it to the context of net-zero transformation^39^.

We propose that NZSC plays a mediating role between DT and EFP for the following reasons: First, DT enables the real-time flow and dissemination of environmental information, helping firms to interpret and respond to external institutional pressures—such as national carbon credit systems and jurisdiction-based climate policies^40^. This accelerates the recognition of net-zero imperatives among decision-makers and enhances the collective understanding of environmental goals. Second, DT fosters cross-functional collaboration and improves internal communication. By breaking down structural silos and encouraging the exchange of heterogeneous knowledge, DT supports the formation of shared beliefs and coordinated actions around sustainability objectives^41,42^. This alignment is critical for effective strategy implementation, particularly when navigating complex and transformative goals like net-zero emissions.

From the Chinese medical equipment manufacturing context, we contend that DT can strengthen enterprises’ NZCS to achieve sustainable competitive goals. Thus, when enterprises instantaneously engaged in DT and net-zero targets, a competitive edge can be attained. Therefore, we state that:

#### H2

 DT has a positive effect on NZSC.

Drawing on the DMC framework, we argue that a management team with strategic consensus is better positioned to sense, seize, and reconfigure resources to drive green innovation and environmental performance. NZSC, as a shared understanding of net-zero strategic goals within the organization, enhances coordination, reduces ambiguity, and enables more effective implementation of sustainability-related decisions.

Prior research has shown that achieving strategic consensus can help enterprises build and reinforce core competitiveness and overall performance^43^. In the context of this study, we contend that NZSC serves as a critical enabler of EFP by guiding both strategic behavior and operational choices toward green and sustainable outcomes. Specifically, NZSC empowers enterprises to accelerate green transformation and capitalize on emerging opportunities within the global green industrial revolution and eco-investment landscape^44^, thereby sustaining the competitiveness of their green products and services.

The influence of NZSC can be manifested in two key ways:

Firstly, from the perspective of workforce awareness and behavior, NZSC helps production managers and employees align with environmental regulations and adopt responsible practices, thus preventing environmental damage caused by negligence or lack of knowledge. Moreover, employee recruitment and training rooted in the principles of NZSC can strengthen environmental awareness and improve competencies related to green production.

Secondly, from the perspective of green production, NZSC is beneficial for decision-makers to purchase more environment friendly raw materials, develop green supply chain, and choose production activities that are more in line with national and international standards. Given the positive impact of supply chain collaboration on environmental performance^45^, NZSC agreement could provide a strong impetus for EFP.

From the perspective of the Chinese medical equipment manufacturing sector, the current study contends that NZSC can influence EFP. Drawing upon the DMC, we posit that NZSC can affect ecological production. Therefore, we propose the following assumptions:

#### H3

NZSC has a positive effect on EFP.

In theory, digital technology is an innovative know-how applied to accumulate, hoard, scrutinize, and share information^46^. Digitization is the application of DT for economic production outcomes^47^. Thus, DT has introduced new processes and mechanisms for key structures in how companies conduct business, aiming to address challenges related to efficiency and effectiveness^48^, which is of great significance for improving business operations, products, processes, and new business models^33,49^. Empirical evidence shows that firms that delay the adoption and execution of digital transformation strategies may struggle to sustain competitiveness in the emerging digital era^50^.

Based on the Resource Based View (RBV), DT is regarded as a strategic resource that can accelerate the integration of information within and outside an organization, and drive the dynamic reconstruction of knowledge^51^. Especially in the context of increasing pressure for green transformation, DT not only enhances the organization’s perception of external sustainable development knowledge, but also enriches the cognitive foundation of managers through the input of heterogeneous information, thereby influencing their attitudes and values towards environmental responsibility and sustainable development^52^.

Furthermore, according to the Dynamic Capability Theory (DMC), when facing external environmental uncertainties such as carbon neutrality policies and green consumption trends, companies must achieve agile response through dynamic restructuring of internal capabilities^53,54^. Among them, NZSC, as a collective cognition and value convergence at the organizational level, reflects the coordination and action orientation of management on green strategic goals. This consensus not only helps companies understand and adapt to changes in environmental policies, but also provides a cognitive foundation and coordination mechanism for the implementation of EFP.

From the perspective of internal knowledge transfer and organizational learning, DT (including big data, cloud computing, artificial intelligence, etc.) provides technical support for enterprises to connect to the global sustainable development knowledge network, enabling effective sharing and dissemination of green concepts and practices within the enterprise^55,56^. This process not only promotes the formation of NZSC, but also strengthens the consistency and execution of the company’s environmental strategy. Reference^57^ found that green outputs such as low-carbon products can serve as mediating variables between DT and firm performance, providing a theoretical precedent for this study. Above all, this study proposes that:

#### H4

 NZSC play a mediating role between DT and EFP.

### Moderating role of SO

DT enables enterprises to rapidly integrate and disseminate internal and external information flows, effectively breaking the constraints of time and space in organizational knowledge acquisition^58^. Through digital technologies, firms can access a broad spectrum of environmental data, policies, and stakeholder expectations, which in turn facilitates the alignment of organizational members around net-zero strategic goals—that is, the formation of NZSC.

However, while DT enhances access to information, the ability to identify, filter, process, and innovate from this complex data stream—especially knowledge related to net-zero strategies—remains a critical challenge^58^. In this regard, SO can be viewed as a critical organizational capability that shapes the extent to which DT facilitates the development of a shared sustainable strategic understanding. Accordingly, SO is expected to play a crucial moderating role in this process.

First, SO refers to a proactive and responsible organizational disposition toward ecological and social issues^20^. It reflects the extent to which environmental values are embedded in managerial cognition and organizational culture. In the context of digital transformation, enterprises are faced with a range of strategic decision-making directions. SO fosters environmental awareness and responsibility among managers^14^, guiding firms to allocate resources toward net-zero objectives during the transformation process. In doing so, SO strengthens the positive effect of DT on NZSC.

Second, SO reflects an enterprise’s commitment to sustainable development. Firms with stronger sustainability awareness are more inclined to leverage digital technologies in developing environmentally friendly products and services^59^. This suggests that SO can amplify the impact of DT on NZSC by enhancing the organization’s capacity to absorb, interpret, and act upon net-zero-related information. In contrast, when SO is low, firms tend to lack the environmental mindset and institutional commitment necessary to fully leverage the sustainability-relevant knowledge enabled by DT. As a result, the potential of DT to foster strategic consensus around net-zero goals is constrained. Conversely, when SO is high, the firm’s environmental consciousness and sense of responsibility are elevated, enabling the aggregation and internalization of net-zero-related knowledge. This, in turn, facilitates the formation of NZSC. Therefore, we state that:

#### H5

 SO positively regulates the linkage amongst DT and NZSC. Enhanced SO enhances the positive impact of DT on NZSC. On the contrary, if SO weakens, then the positive impact of DT on NZSC weakens.

### The moderation mediating effect

SO can significantly shape organizational attitudes towards environmental protection policies and green development strategies^20^. It functions as a strategic filter that channel digital technology resources towards advancing net-zero strategies and environmental protection initiatives.

On the one hand, enterprises with high level SO are more adept at leveraging digital green capabilities to transform production data into actionable carbon reduction plans, thereby achieving superior environmental performance compared to those with low SO^60^. In the context of medical equipment enterprises, SO facilitates the allocation of resources toward net-zero strategies during digital transformation, thereby promoting EFP. Consequently, as SO strengthens, the mediating effect of NZSC becomes more significant.

On the other hand, enterprises with a high level of strategic orientation are more inclined to embed carbon neutrality into their core strategies^61^. NZSC serves as both a cognitive foundation and a coordination mechanism at the organizational level. When SO is strong, the knowledge flow facilitated by DT is more effectively absorbed and converted into a shared strategic consensus, thereby amplifying the downstream impact of NZSC on EFP. Hence, we propose that:

#### H6

The enhanced SO, the stronger the mediating effect of NZSC in the model.

## Methods

This study utilized SPSS 26 and PROCESS v2.13.2 for regression analysis. SPSS was employed for descriptive statistical analysis, reliability analysis, exploratory factor analysis, and regression analysis. PROCESS was employed for mediator bootstrap analysis and drawing the moderating effect diagram.

**Fig. 2:**
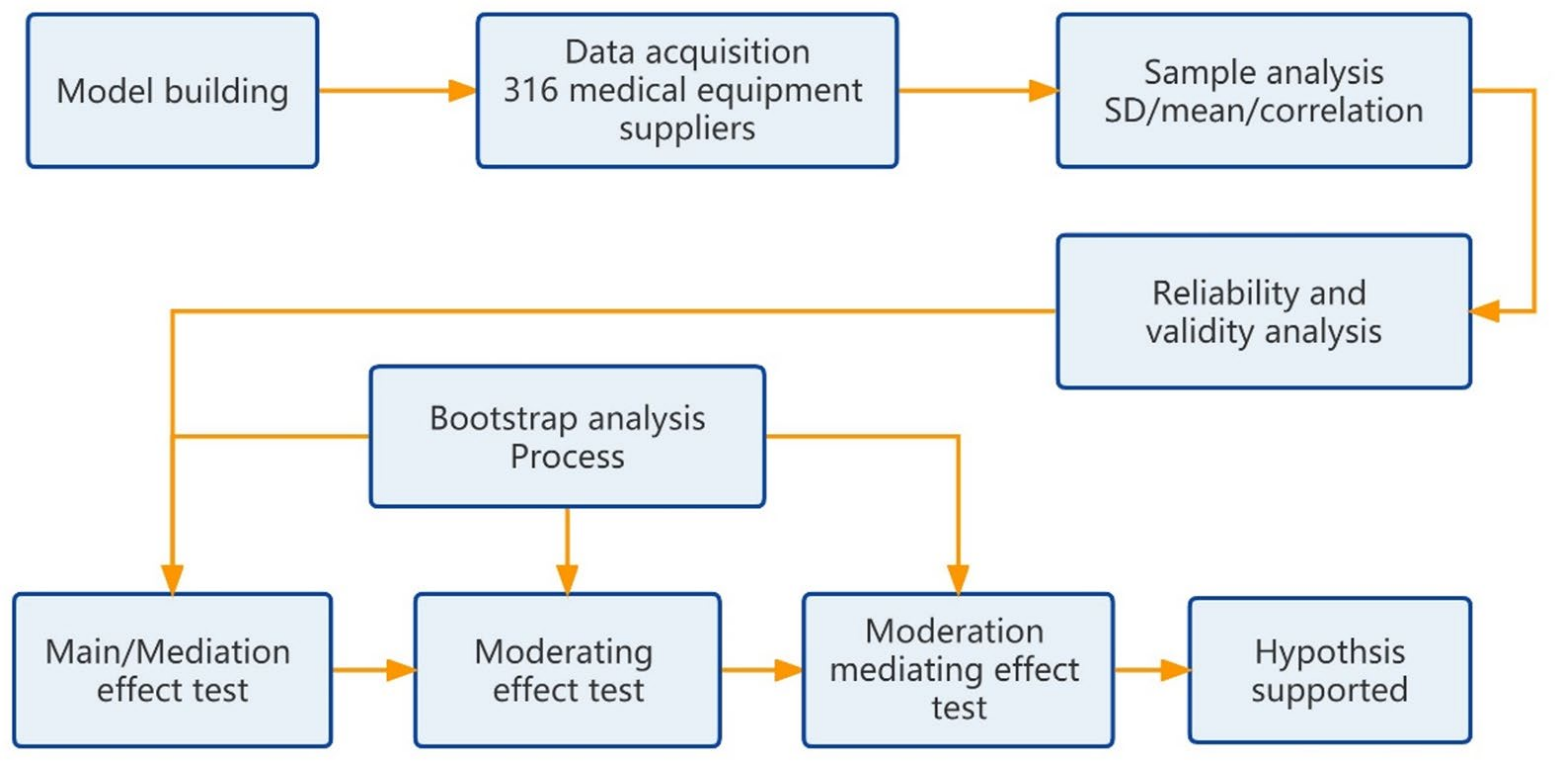
Flow chart of the study.

The method of this study is shown in Fig. 2 (Flow chart).

### Model building

Given the research framework formulation, our regression models are as follows:

M1: *EFP* = *β*_*0*_ *+ β*_*1*_ *Age + β*_*2*_ *Size + β*_*3*_ *Ownership + β*_*4*_ *R&D + β*_*5*_ *Subsidy + β*_*6*_ *Net P + µ*.

M2: *EFP* = *β*_*0*_ *+ β*_*1*_ *Age + β*_*2*_ *Size + β*_*3*_ *Ownership + β*_*4*_ *R&D + β*_*5*_ *Subsidy + β*_*6*_ *Net P + β*_*7*_ *DT + µ*.

M3: *EFP* = *β*_*0*_ *+ β*_*1*_ *Age + β*_*2*_ *Size + β*_*3*_ *Ownership + β*_*4*_ *R&D + β*_*5*_ *Subsidy + β*_*6*_ *Net P + β*_*7*_ *NZSC + µ*.

M4: *EFP* = *β*_*0*_ *+ β*_*1*_ *Age + β*_*2*_ *Size + β*_*3*_ *Ownership + β*_*4*_ *R&D + β*_*5*_ *Subsidy + β*_*6*_ *Net P + β*_*7*_ *DT + β*_*8*_ *NZSC + µ*.

M5: *NZSC* = *β*_*0*_ *+ β*_*1*_ *Age + β*_*2*_ *Size + β*_*3*_ *Ownership + β*_*4*_ *R&D + β*_*5*_ *Subsidy + β*_*6*_ *Net P + µ*.

M6/M7: *NZSC* = *β*_*0*_ *+ β*_*1*_ *Age + β*_*2*_ *Size + β*_*3*_ *Ownership + β*_*4*_ *R&D + β*_*5*_ *Subsidy + β*_*6*_ *Net P + β*_*7*_ *DT + µ*.

M8: *NZSC* = *β*_*0*_ *+ β*_*1*_ *Age + β*_*2*_ *Size + β*_*3*_ *Ownership + β*_*4*_ *R&D + β*_*5*_ *Subsidy + β*_*6*_ *Net P + β*_*7*_ *DT + β*_*8*_ *SO + µ*.

M9: *NZSC* = *β*_*0*_ *+ β*_*1*_ *Age + β*_*2*_ *Size + β*_*3*_ *Ownership + β*_*4*_ *R&D + β*_*5*_ *Subsidy + β*_*6*_ *Net P + β*_*7*_ *DT + β*_*8*_ *SO + β*_*9*_ $\:\stackrel{-}{DT}$ * $\:\stackrel{-}{SO}$ *+µ*.

### Data acquisition

This study was approved by the ethics committee of Dazhou Central Hospital, and all methods were performed in accordance with the relevant guidelines and regulations. At the same time, this study explained the purpose of obtaining these data to the respondents before the questionnaire survey, and only collected the data after obtaining their consent. We state that the informed consent was obtained from all subjects and/or their legal guardian(s).

This study selected medical device enterprises within China as the survey subjects. Data collection consisted of a pre-survey and formal survey.

**Pre-survey.** First, this study invited 10 executives to participate in interviews to identify potential issues or suggestions related to the questionnaire. Based on their feedback, modifications were made to ambiguous item (e.g., difficult-to-understand statements, lack of compliance with industry standards, or vague wording from respondents’ perspectives) to better align with corporate realities and enhance clarity. Additionally, attention-check items were incorporated to ensure data authenticity and reliability. Second, a pre-survey was conducted to further identify design issues and improve the questionnaire through small-sample data collection. For feasibility, this study leveraged collaborative research projects to distribute pre-study questionnaires to 39 medical device suppliers affiliated with two large hospitals (each with annual revenue exceeding 1 billion yuan). The pre-survey results show that the KMO values for DT, NZSC, SO, EFP are 0.792, 0.747, 0.755, 0.894, respectively- all greater 0.7. Similarly, the Cronbach’s alpha coefficient of DT, NZSC, SO, EFP are 0.915, 0.903, 0.951, respectively, all exceeding 0.7, thereby indicating good reliability and validity of the questionnaire.

**Formal survey and data collection.** This study relied on cooperative projects with multiple tertiary hospitals and school-enterprise collaboration platforms to distribute questionnaires to the heads of medical device suppliers listed on hospital supplier rosters. These suppliers are widely distributed across China. The data collection period spanned from January 2023 to March 2023. Of the 409 questionnaires distributed, 329 were collected. After screening and eliminating 13 invalid questionnaires suspected of response bias, 316 valid samples were retained, yielding a valid response rate of 77.26%.

### Measure

(1) Dependent variable: Environmentally friendly production.

Referring to the study by Almaqtari, et al.^1^, this study defines EFP as a production activity that considers environmental factors in product design, material selection, production process, consumer payment, and scrap management and forms five measurement items based on this.

(2) Independent variable: Digital transformation.

Digital transformation is a change activity executed by enterprises using digital transformation^62^. These activities include but are not limited to big data, artificial intelligence, cloud computing, mobile and social media platforms, and other technologies for new business development, process simplification, customer experience improvement, etc.

Referring to Vial’s research on Digital transformation, this study has formed the following measurement items: in the past three years, digital technology has been used for organizational structure and cultural transformation. In the past three years, utilizing digital technology to drive changes in leadership style, employee roles, and skills; In the past three years, utilizing digital technology to develop new business processes or sales channels; In the past three years, digital technology has been used to quickly adapt to environmental changes.

(3) Mediating variable: NZSC.

Hakovirta, et al.^39^ point out that the goal of achieving net-zero emissions—or carbon neutrality—refers to the realization of net-zero carbon dioxide emissions in a company’s operations and its broader environmental footprint. Achieving this goal requires the development of essential solutions, including both the reduction of greenhouse gas emissions associated with societal needs and the removal of carbon dioxide from the atmosphere. According to Yao, et al.^38^, strategic consensus is primarily reflected in the extent to which organizational members align with the following three dimensions: the long-term strategic goals of the company, the short-term business objectives, and the best ways to ensure the company’s survival. Building on these foundations, this study defines NZSC as the degree to which organizational members share a common understanding and agreement regarding the company’s net-zero strategy and its related goals. Accordingly, the following measurement items are proposed: Our company members have consistency in the long-internet-zero strategic goals of the organization; Our company members have consistency in the short-term net zero business goals of the organization; Our company members believe that the organization’s net zero strategies are beneficial for the long-term survival of the company.

(4) Moderating variable: Sustainability orientation.

SO reflects an organization’s capacity to identify and exploit entrepreneurial opportunities in ways that are both ecologically and socially responsible^12^. As such, SO has gained increasing strategic relevance for firms, as its benefits extend beyond enhancing corporate image to improving operational efficiency^20^. Given these advantages, SO is viewed as a firm-level strategic construct that encourages companies to recognize, engage with, and commit to environmental issues, initiatives, and long-term programs^14^. Managers are therefore expected to embed SO into the organization’s philosophy, culture, and strategic direction to guide its operational priorities^14^.

Referring to Danso, et al.^20^, SO can be measured through three core dimensions: knowledge, practices, and commitment toward sustainability. Based on this framework, and considering the actual context of Chinese enterprises, we have adapted and developed the following three measurement items: (1) Employees have learned sustainability knowledge related to their positions and are familiar with sustainability related projects; (2) Sustainability is an important part in our daily work; (3) Sustainability practices are good for our company and relevant actions will be carried out for a long time.

(5) Control variables.

Considering the influence of sample characteristics and differences, this article refers to the research of Xie, et al.^63^ and controls for the age, size, ownership, R&D intensity, government subsidies, and net profit of enterprises. Among them, due to the independent variable considering the changes in the past three years, the surveyed enterprises in this study are all older than 3 years. The size of the enterprise is measured by the number of employees; ownership includes state-owned enterprises (represented by 1) and private enterprises (represented by 0); we measure R&D intensity using the proportion of R&D investment to sales revenue.

The formal questionnaire is shown in appendix, and references for item design are shown in Table 1.

**Table 1:** The references for item design.

Variable	References
EFP	Almaqtari, et al. ^1^
DT	Xie, et al. ^62^
NZSC	Hakovirta, et al. ^39^; Yao, et al. ^38^
SO	Danso, et al. ^20^

## Empirical analysis

### Common method bias test

Common method bias (CMB) is a significant concern in survey-based studies. It refers to the systematic variance shared among variables in a study due to the measurement method itself rather than the actual relationships between the constructs being measured. Firstly, this article uses Harman’s single-factor test to examine the sample data for common method bias. It imports all data into SPSS for factor analysis and selects eigenvalues greater than 1. The conclusions indicate that the contribution rate of the first factor is 33.25% (reference standard is less than 40%), and the cumulative contribution rate of the top five factors is 67.52% (reference standard is greater than 60%). This indicates no serious common method deviation in the sample data. Secondly, referring to Chin, et al.^64^, this study employed the common marker variable technique to strengthen the validity of the findings. Given that respondents’ gender, age, and education level have no theoretical association with the core constructs of the model, these demographic variables were selected as marker variables. Two models were constructed for comparison. As shown in Table 2, Model 1 constrained all factor loadings from the common method factor to zero, assuming the absence of common method bias (CMB), and yielded a chi-square value of 234.017 with 126 degrees of freedom. In contrast, Model 2 allowed all factor loadings from the common method factor to be freely estimated, resulting in the same chi-square value of 234.017 but with 125 degrees of freedom. The resulting chi-square difference between the two models was 0 (Δdf = 1), which was not statistically significant (*p* > 0.05). This result indicates that CMB is unlikely to pose a serious threat to the validity of the study’s findings.

**Table 2:** Estimation results of common method bias.

	Model 1	Model 2
Chi square	234.017	234.017
Degrees of freedom	126	125

### Reliability and validity

This article used Cronbach’s α coefficient to evaluate the reliability of the survey. The conclusions in Table 3 indicate that Cronbach’s correspond to DT, NZSC, SO, and EFP. The coefficients are 0.919, 0.929, 0.900, and 0.925, respectively; thus, all the values are > the reference score of 0.6. This indicates the good reliability of the questionnaire data in this study.

This paper evaluates the correlation between latent and observable variables and the combined validity indicators. The data analysis conclusions in Table 1 demonstrate that the KMO values corresponding to DT, NZSC, SO, and EFP are 0.855, 0.765, 0.746, and 0.853, respectively, which are all greater than the reference value of 0.6; The AVE values corresponding to each variable are 0.804, 0.876, 0.833, 0.771, all > the reference score of 0.5; The CR values corresponding to each variable are 0.943, 0.955, 0.937, and 0.944, all > the reference score of 0.7. This indicates a good correlation between the variables in this study, and the data has good combinatorial validity.

Furthermore, this study conducts HTMT analysis to test the discriminant validity of the questionnaire. The results show that the maximum HTMT coefficient in the matrix is 0.555, which is less than 0.85; the bootstrap 95% CI interval does not contain 1. The result indicates that this study has good discriminant validity.

**Table 3:** Reliability and validity analysis.

Valuables	KMO	Factor Loading	AVE	CR	Cronbach’s α
DT	0.855	0.901	0.804	0.943	0.919
		0.873			
		0.902			
		0.910			
NZSC	0.765	0.941	0.876	0.955	0.929
		0.929			
		0.937			
SO	0.746	0.914	0.833	0.937	0.900
		0.926			
		0.897			
EFP	0.853	0.898	0.771	0.944	0.925
		0.914			
		0.802			
		0.912			
		0.860			

Finally, we conducted a confirmatory factor analysis on 316 research data using AMOS software to assess whether the six core variables of DT, NZSC, SO and EFP met the requirements. Table 4 shows that among all models, the 4-factor model has the best fitting index. Specifically, the following fit indices were observed: χ2/df = 2.356 (< 3), RMSEA = 0.066 (< 0.08); SRMR = 0.0334 (< 0.05), IFI = 0.970, RFI = 0.936, TLI = 0.962, GFI = 0.921, NFI = 0.949, CFI = 0.970, all of which were above the recommended value of 0.9. The results indicate that the model fit of this study is ideal, and the questionnaire scale demonstrates good structural validity.

**Table 4:** Confirmatory factor analysis.

Type	IFI	RFI	TLI	GFI	NFI	CFI	x^2^/df	RMSEA	SRMR
Baseline model (4 factors)	0.970	0.936	0.962	0.921	0.949	0.970	2.356	0.066	0.0334
3 factors model	0.963	0.930	0.952	0.922	0.946	0.963	3.104	0.082	0.0336
2 factors model	0.953	0.919	0.934	0.912	0.942	0.952	5.169	0.115	0.0388
1 factors model	0.918	0.829	0.836	0.873	0.915	0.918	22.578	0.262	0.0503

**Table 5:** Mean, SD and correlation analysis.

Variables	Firm age	Firm size	Ownership	RD	FS	Net profit	DT	NZSC	SO	EFP
Firm age	1									
Firm size	0.082	1								
Ownership	0.002	0.206**	1							
RD	0.070	0.155**	0.183**	1						
FS	0.080	0.149**	0.166**	0.118*	1					
Net profit	0.054	0.161**	0.192**	0.116*	0.158**	1				
DT	0.052	0.341**	0.241**	0.192**	0.180**	0.318**	1			
NZSC	-0.002	0.327**	0.318**	0.259**	0.257**	0.350**	0.433**	1		
SO	0.043	-0.079	0.052	0.099	0.068	0.108	0.024	0.029	1	
EFP	-0.011	0.341**	0.271**	0.306**	0.319**	0.276**	0.409**	0.514**	0.088	1
Mean	2.712	2.877	0.345	2.886	2.826	3.203	3.993	3.938	4.214	4.072
SD	1.348	1.405	0.476	1.396	1.366	1.418	1.811	1.920	1.742	1.616

Remark: *N* = 316; * *p* < 5%, ** *p* < 1%.

**Table 6:** Regression analysis for main and mediating effects.

Variables	Dependent variable: EFP				Dependent variable: NZSC	
	Model 1	Model 2	Model 3	Model 4	Model 5	Model 6
Firm age	-0.071(0.058)	-0.073(0.056)	-0.053(0.055)	-0.056(0.054)	-0.055(0.069)	-0.057(0.067)
Firm size	0.231(0.058) ***	0.174(0.058) ***	0.162(0.056) ***	0.131(0.057) **	0.211(0.068) ***	0.150(0.069) **
Ownership	0.118(0.171) *	0.092(0.168)	0.061(0.165)	0.050(0.164)	0.175(0.204) ***	0.148(0.199) **
RD	0.209(0.057) ***	0.187(0.056) ***	0.159(0.055) ***	0.150(0.054) **	0.152(0.068) **	0.129(0.066) **
FS	0.220(0.059) ***	0.204(0.057) ***	0.173(0.056) ***	0.167(0.056) ***	0.145(0.07) **	0.127(0.068) **
Net profit	0.161(0.057) **	0.109(0.057) *	0.081(0.056)	0.055(0.056)	0.244(0.067) ***	0.188(0.067) ***
DT		0.224(0.047) ***		0.155(0.047) **		0.242(0.056) ***
NZSC			0.328(0.045) ***	0.287(0.046) ***		
R2	0.276	0.314	0.352	0.368	0.273	0.317
F	21.033	21.595	25.452	23.922	20.721	21.923
Max VIFs	1.111	1.282	1.402	1.498	1.111	1.282

Remark: *N* = 316, ***p<1‰, **p<1%, *p<5%. β (standard error).

### Descriptive statistics and correlation analysis

Table 5 reports the correlation between the mean, standard deviation, and variables. The results show that all correlation coefficients < 0.7, DT is positively correlated with EFP (*p* < 0.01), DT is positively correlated with NZSC (*p* < 0.01), and NZSC is positively correlated with EFP (*p* < 0.01), which is basically in line with the prediction of the model in this article. Ownership and R&D intensity significantly impact EFP, which is consistent with the view of existing studies^65,66^and further proves that our model has good robustness.

The K-S test results indicate that the asymptotic significance values of DT and EFP are less than 0.05. Furthermore, based on the mean and standard deviation distribution in Table 5, it can be concluded that the samples do not follow a normal distribution and primarily composed of small and medium-sized enterprises. Further, we test the VIF value to determine if there are serious multicollinearity issues, generally requiring a value less than 5. The maximum VIF values in all regression models are < 5, so serious collinearity interference is excluded.

### Regression analysis

#### Main effect model and mediating effect model

Table 6 reports the main effect and Mediating effect regression results of this study.

Model 1 is a regression model of the control variable on EFP. The data shows that factors such as firm size, ownership, RD, FS, and net profit have a positive association with EFP, which is in line with the basic situation of the industry.

Model 2 is a regression model that adds independent variables to Model 1. Data shows that DT has a positive relationship with EFP(β = 0.224, *p* < 0.001), H1 is supported.

Model 3 is the regression model of NZSC for EFP, while Model 4 controls DT based on Model 3.

Model 4 results show that NZSC has a positive relationship with EFP (β = 0.287, *p* < 0.001), H3 is supported.

Model 5 is a regression model of the control variable on NZSC. Model 6 has added the independent variable DT on top of Model 5. The results show that DT has a positive relationship with NZSC( β = 0.242, *p* < 0.001), H2 is supported.

The results in Table 6 show that all VIFs are below 3.0, which is well below the generally accepted threshold of 5, indicating that collinearity is unlikely to affect the stability of the results^67^.

Based on the results of M1-M6, DT has a positive effect on NZSC, NZSC has a positive impact on EFP, and DT has a positive impact on EFP. According to the definition of the Mediating effect, we can infer that NZSC plays a partial mediation role in DT and EFP.

Furthermore, this paper uses PROCESS to conduct bootstrap analysis to test the stability of the mediating effect. Table 7 shows that the direct effect of model DT-NZSC-EFP is 0.1383, accounting for 69.12%, confidence interval is [0.0462, 0.2303], excluding 0; The indirect effect is 0.0618, accounting for 30.88%, confidence interval is [0.0293, 0.1039], excluding 0; The total effect is 0.2000, and the confidence interval does not include 0. This indicates that NZSC partially mediates the DT-EFP relationship, and the results have a certain degree of robustness. H4 is supported.

**Table 7:** Bootstrap analysis of the mediating effect of NZSC.

Item	Coeff	S. E	CI = 95%, 5000 times		Rate
			LLCI	ULCI	
Total	0.2001	0.0471	0.1074	0.2929	100%
Direct	0.1383	0.0468	0.0462	0.2303	69.12%
Indirect	0.0618	0.0190	0.0293	0.1039	30.88%

#### Moderating effect of SO

Before conducting regression analysis, this study dispersed the interaction terms using the following formula: (independent variable minus mean of independent variable) multiplied by (moderating variable minus mean of moderating variable), and finally ran SPSS to obtain Table 8. Model 9 results show that the interaction between DT and SO has a positive relationship with NZSC (β = 0.179, *p* < 0.001), indicating a significant regulatory effect of SO between DT and NZSC.

**Fig. 3:**
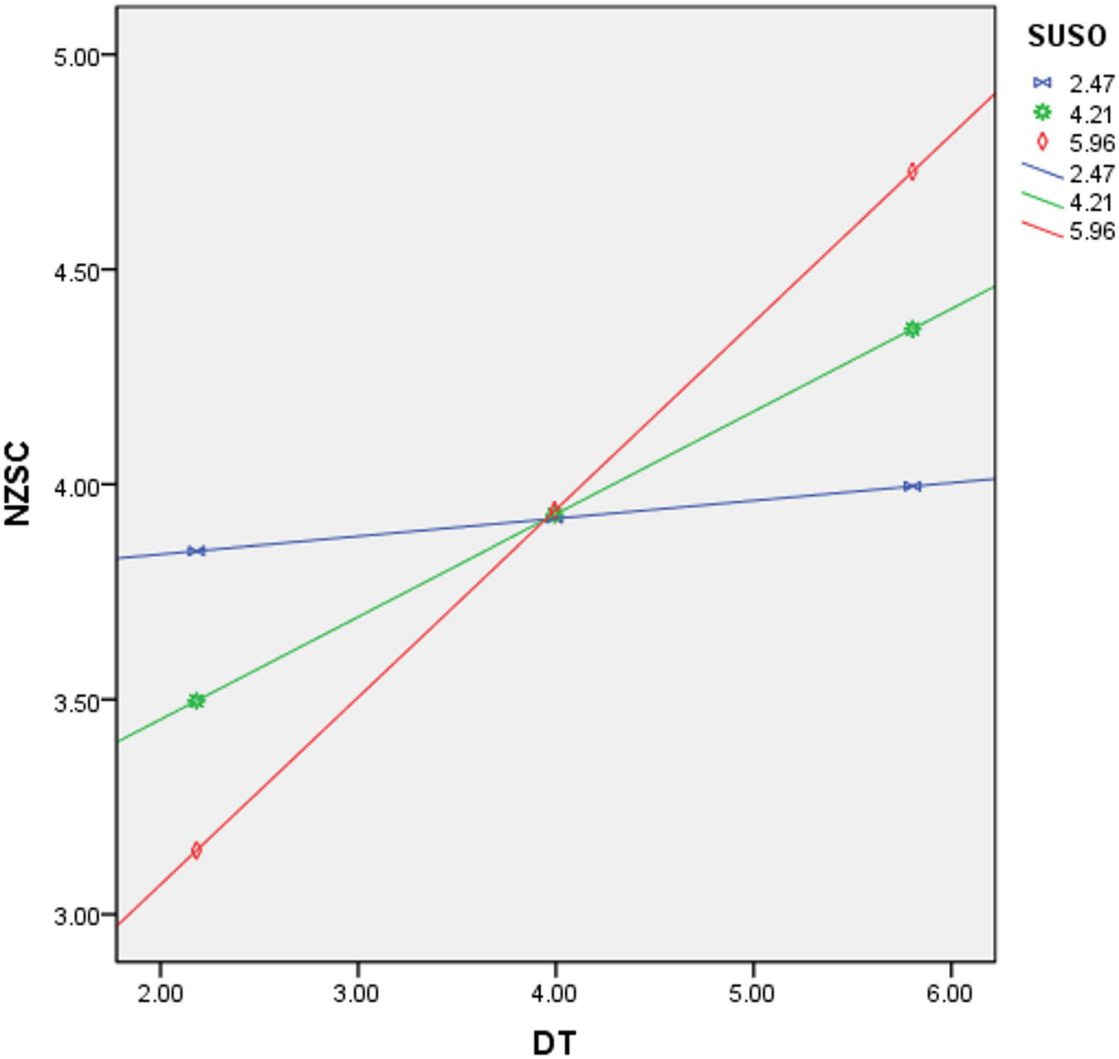
Moderating effect of SO.

Furthermore, in order to demonstrate the role of SO more clearly, this study drew a regulatory effect diagram. Figure 3 shows that when SO is at a lower level, the promoting effect of DT on EFP is gentle, and the slope is small. When SO is at a high level, the promoting effect of DT on EFP is enhanced, and the slope significantly increases. This further indicates that SO has a significant regulatory effect in the model, H5 is supported.

**Table 8:** Moderating effects of NZSC.

Variables	Dependent variable: NZSC		
	Model 7	Model 8	Model 9
Firm age	− 0.057(0.067)	− 0.056(0.067)	− 0.051(0.066)
Firm size	0.150(0.069) **	0.148(0.069) **	0.134(0.068) **
Ownership	0.148(0.199) **	0.148(0.2) **	0.148(0.196) **
RD	0.129(0.066) **	0.130(0.067) **	0.122(0.065) *
FS	0.127(0.068) **	0.127(0.068) **	0.116(0.067) *
Net profit	0.188(0.067) ***	0.190(0.068) ***	0.166(0.067) ***
DT	0.242(0.056) ***	0.242(0.056) ***	0.225(0.055) ***
SO		− 0.013(0.052)	0.005(0.052)
DT*SO			0.179(0.030) ***
R2	0.317	0.315	0.344
F	21.923***	19.133***	19.342***
Max VIFs	1.282	1.282	1.291

Remark: *N* = 316, ***p<1‰, **p<1%, *p<5%. β (standard error).

#### Moderated mediation effect model

**Fig. 4:**
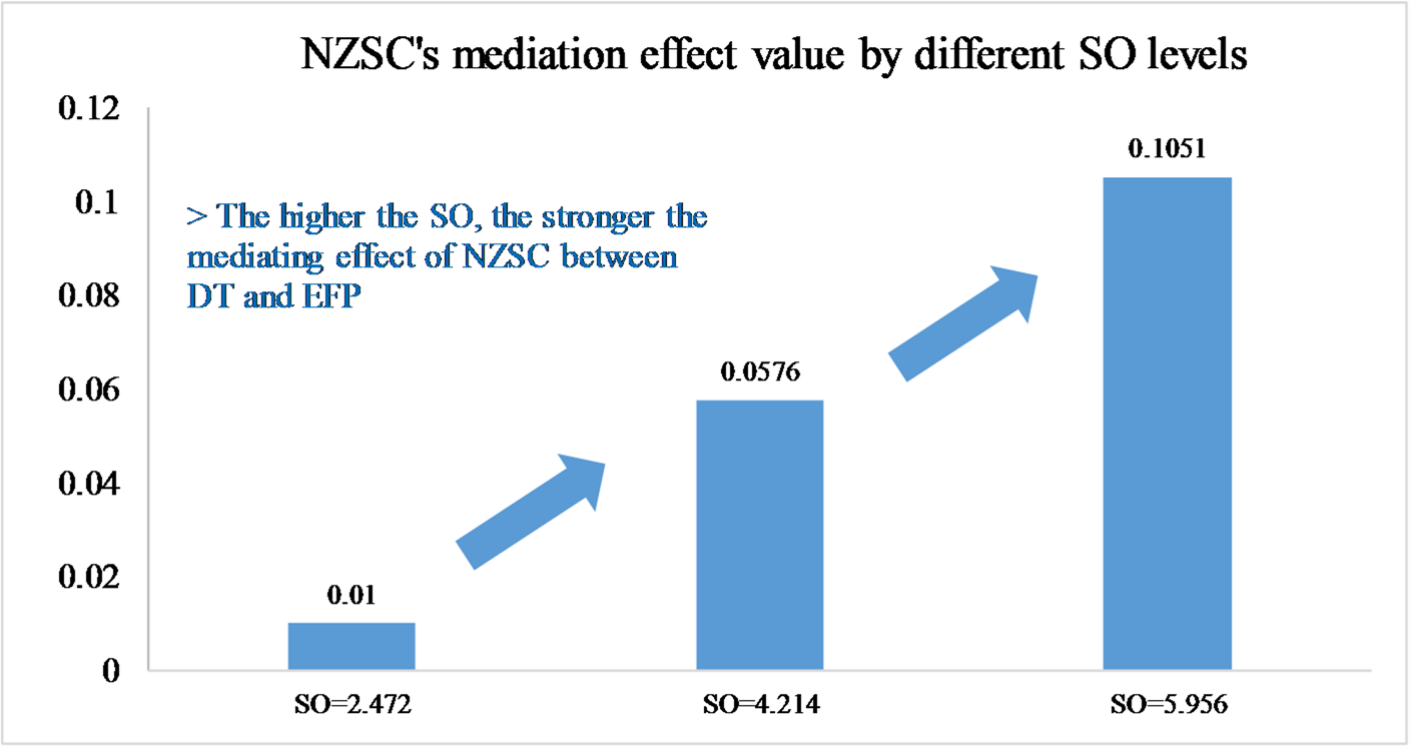
Mediation effect value by different SO levels.

This study conducted the moderated mediation effect model analysis using PROCESS software, see Table 9. The data shows that when SO is at a low level, the confidence interval contains 0, and then the mediating effect of NZSC becomes insignificant. Table 9; Fig. 4 show that with the increase of SO, the mediating effect coefficient of NZSC gradually increases and becomes significant (the confidence interval does not contain 0). Overall, the mediation effect coefficient of the study is 0.0273, confidence interval is [0.0126, 0.0485], excluding 0. These indicators indicate that the moderated mediation effect of SO is significantly present, H6 is supported.

**Table 9:** The moderating mediation effects.

Moderator	SO	Coeff	S. E	LLCI	ULCI
NZSC	2.4722	0.0100	0.0206	− 0.0318	0.0517
	4.2141	0.0576	0.0181	0.0274	0.0985
	5.9561	0.1051	0.0272	0.0577	0.1661
Moderated mediation effect	N/A	0.0273	0.0092	0.0126	0.0485

## Discussion and implications

### Conclusions

The findings reveal that: (1) DT has a positive impact on EFP; (2) DT facilitates EFP by promoting the mediating variable NZSC; (3) SO positively regulates the linkage between DT and NZSC; (4) The enhanced SO, the stronger the mediating effect of NZSC in the model. This study reveals the effects, mediating mechanism, and moderating mechanism of DT on EFP, which is beneficial for providing important contribution and insights for related theoretical research, policy formulation, and green development of enterprises.

### Theoretical contributions

This study significantly expands the existing research by providing a more comprehensive understanding of how Chinese medical equipment suppliers leverage DT to create environmental value and achieve competitive advantage.

Based on literature review, scholars have conducted in-depth exploration on the relationship between DT and EFP. Existing research has confirmed the positive impact of DT on EFP from the perspectives of digital green capability^60^, green supply chain collaboration^57^, and green innovation^68^. Compared with previous studies, this study starts from the perspective of strategic consensus and confirms that DT can shape NZSC and further enhance EFP by promoting the concentration and dissemination of net zero strategic information, enriching the research on the impact path of DT on EFP.

Furthermore, prior studies seriously lacks attention to relationships between DT and overall firm performance^42^. This study delves into the internal mechanism linking DT and EFP, a crucial step beyond merely establishing a correlation. Previous studies have used Resource-Based View (RBV) and Dynamic Managerial Capabilities (DMC) theories to analyze DT and sustainability^5,69^, while our research integrates these frameworks to explain how DT as valuable organizational resources interact with management cognition and collaboration to promote EFP. We particularly emphasize that the strategic consensus on the net zero target is a key but yet to be fully explored mediating variable in this process. Thus, our research provides a novel theoretical perspective that links DT with environmental behavior through internal strategic synergy, thereby expanding the application of RBV and DMC in the context of sustainable development.

While net-zero goals have become central to national and corporate sustainability agendas, there remains a significant lack of empirical research that investigates how net-zero strategic consensus within firms influences their operational sustainability outcomes, such as EFP. Previous studies have focused on the impact of technological tools such as artificial intelligence or Geographic Information Systems on the implementation of net zero policies^70^, but have overlooked the role of organizational capacity. This study considers NZSC as a dynamic capability and confirms its mediating role in the relationship between DT and EFP. Driven by DT, NZSC helps to adjust and change the resources, processes, and structure that enable companies to participate in EFP. This finding deepens people’s understanding of how DT promotes environmental behavior in practice.

Lastly, the research emphasizes the moderating effect of sustainability orientation (SO). From the digital economy perspective, the internal and external information flow gathered by DT can accelerate the achievement of NZSC. Such information is shallow, fragmented, and diverse^71,72^, but if misleading information cannot be effectively identified and utilized, it may hurt the environment. Therefore, how to efficiently identify, screen, and integrate this seemingly rich but scattered environmental protection-related knowledge has become a critical challenge^58^. Existing research mostly focuses on the impact of SO on firm performance, lacking in-depth exploration of the influence of SO on organizational capacity building ^12,73^. This study considers SO as a key green strategic resource, demonstrating its scarcity and value, as well as enhancing the ability of enterprises to identify and integrate environmental information, thereby promoting the formation of net zero consensus. This perspective enriches the understanding of the RBV on how resources play a role in green transformation. In addition, this study expands the application boundaries of DMC theory in the field of green digital transformation. This study suggests that SO is not only a resource, but also a ‘capability’—helping businesses quickly respond, integrate, and transform complex and fragmented environmental information into sustainable strategic actions.

### Implications

By understanding the relationships between DT, NZSC, SO, and EFP, policymakers can create a supportive environment that encourages businesses to embrace sustainable practices, leading to positive environmental and economic outcomes.

First, our findings confirm that DT significantly drives EFP. The medical device industry is subject to strict product safety, quality certification (such as NMPA) and environmental regulations, which to a certain extent limits the ability of enterprises to quickly trial and error in environmental protection technologies and systems. Therefore, the government should reduce the risks and costs of enterprises in promoting DT under the premise of compliance through green approval channels, green compliance guidelines for digital technology, and tax deduction policies. For example, special funding support or green credit policies can be given to smart production lines that reduce energy consumption and waste emissions through digital means.

Second, NZSC facilitates the restructuring of organizational processes toward sustainable outcomes. Governments can promote NZSC by organizing awareness campaigns, funding industry knowledge-sharing platforms, and encouraging cross-sector net-zero partnerships. Managers, in turn, should establish regular training mechanisms to build NZSC and ensure alignment between digital tools and long-term sustainability commitments.

Third, SO strengthens the pathway from DT to EFP through NZSC. Given its amplifying effect, policymakers can Integrate SO metrics into digital transformation evaluation frameworks, ensuring that sustainability values are assessed alongside efficiency and innovation. Plus, government should support the development of sustainability leadership programs tailored for medical device firms, focusing on how managers can embed environmental values in digital strategy development. Government can also offer consulting subsidies or toolkits to help SMEs in this sector embed sustainability into their digital planning and implementation processes. Firms, in turn, should institutionalize sustainability values by embedding SO into performance evaluation systems, leadership development pipelines, and digital project approval frameworks. For example, companies can require that all digital investment proposals include a sustainability impact assessment and provide training for IT and operations teams on environmental responsibility.

Finally, the proposed framework can be adapted across industries to guide the design and evaluation of digital sustainability initiatives, offering a practical roadmap for achieving net-zero goals through digital innovation.

### Limitations

First, the data are limited to the medical field and enterprise level. The Chinese medical equipment manufacturing firms has unique cultural context and industry characteristics and regulatory frameworks that may influence the applicability of findings to other sectors. This study focuses on firms within the Chinese medical equipment industry, and while the sample covers a broad range of firms, it is not based on a nationally representative sampling framework. This may limit the generalizability of the findings and should be addressed in future research with larger and more representative samples. The study’s emphasis on the mediating role of NZSC in the relationship between DT and EFP could be applicable to other sectors. Achieving consensus on net-zero goals and strategies can be crucial for driving sustainable practices in any industry. The future studies can extend to other industries and fields, potentially leading to more comprehensive and widely applicable conclusions;

Second, limitations of data and methods. This study takes medical equipment companies as samples, which helps to focus on industry characteristics, but the research conclusions still need to be cautiously validated when extended to other industries. Future research can validate the theoretical framework and impact mechanism proposed in this paper in a wider range of industries. Although the response rate of 77.26% is relatively high for an executive-level survey, the possibility of nonresponse bias cannot be completely ruled out. Nonresponse may stem from time constraints or availability issues rather than differences in organizational characteristics. However, this remains a potential limitation that future research could address more systematically.

Third, the effects of DT on EFP may be mediated by variables other than NZSC. For example, a company’s green organizational culture, employees’ environmental awareness, and the degree of green collaboration in the supply chain may all play a mediating role between DT and EFP. Meanwhile, NZSC is not confined to RBV and DMC perspectives, and alternative interpretations may emerge under different theoretical lenses and research frameworks. Therefore, future research could further expand the examination of mediating pathways and explore additional structural mechanisms or theoretical perspectives to provide a more comprehensive understanding of how digitalization strategies drives the realization of EFP through multiple channels.

## Supplementary Information

Below is the link to the electronic supplementary material.

Supplementary Material 1

## Data Availability

Data is provided within the manuscript or Supplementary files.
