# Supplementary material for: Promoting Chinese medical equipment enterprises’ environmentally friendly production through digital transformation and net zero strategic consensus
Source: Sci Rep. 2025 Nov 27;15:42363. doi: 10.1038/s41598-025-26285-6 (PMC12660354; doi:10.1038/s41598-025-26285-6)
Supplement: Supplementary file 1 — Supplementary Material 1 [file 41598_2025_26285_MOESM1_ESM.pdf]

NO	A1	A2	A3	A4	A5	A6	DT	NZSC	SUSO	EFP	Q1	Q2	Q3	Q4	Q5	Q6	Q7	Q8	Q9	Q10	Q11	Q12	Q13	Q14	Q15
1	5	4	0	1	3	4	4.50	2.33	1.67	3.00	2.00	5.00	5.00	6.00	3.00	2.00	2.00	2.00	2.00	1.00	3.00	2.00	2.00	3.00	5.00
2	1	2	0	4	2	2	1.25	3.33	3.00	5.80	2.00	1.00	1.00	1.00	1.00	5.00	4.00	3.00	3.00	3.00	6.00	5.00	5.00	7.00	6.00
3	1	1	0	5	1	5	2.75	3.67	6.00	2.20	3.00	3.00	3.00	2.00	3.00	4.00	4.00	4.00	7.00	7.00	2.00	2.00	2.00	2.00	3.00
4	4	2	0	3	1	2	3.25	2.33	4.00	2.20	3.00	7.00	2.00	1.00	2.00	2.00	3.00	5.00	4.00	3.00	3.00	3.00	1.00	2.00	2.00
5	1	3	0	4	3	1	3.00	4.00	2.00	5.00	2.00	5.00	2.00	3.00	3.00	5.00	4.00	2.00	2.00	2.00	4.00	5.00	6.00	4.00	6.00
6	1	1	1	5	2	5	4.25	5.00	2.33	3.60	6.00	5.00	5.00	1.00	5.00	5.00	5.00	3.00	2.00	2.00	1.00	4.00	5.00	3.00	5.00
7	4	2	0	2	2	1	2.00	1.33	6.33	1.80	3.00	3.00	1.00	1.00	2.00	1.00	1.00	7.00	7.00	5.00	2.00	2.00	2.00	1.00	2.00
8	4	3	0	5	2	3	2.25	2.67	3.67	5.40	1.00	3.00	2.00	3.00	3.00	2.00	3.00	3.00	4.00	4.00	5.00	7.00	5.00	5.00	5.00
9	3	1	0	1	3	1	2.50	1.33	6.00	5.40	3.00	3.00	2.00	2.00	2.00	1.00	1.00	7.00	5.00	6.00	5.00	6.00	5.00	6.00	5.00
10	3	2	0	5	3	3	5.00	6.00	5.33	5.80	4.00	7.00	4.00	5.00	6.00	7.00	5.00	5.00	6.00	5.00	5.00	5.00	7.00	6.00	6.00
11	2	5	1	5	2	5	1.50	4.33	6.67	5.20	1.00	2.00	2.00	1.00	4.00	4.00	5.00	7.00	7.00	6.00	5.00	5.00	4.00	6.00	6.00
12	2	1	0	3	4	2	2.00	1.33	5.00	5.40	2.00	1.00	2.00	3.00	2.00	1.00	1.00	6.00	6.00	3.00	6.00	5.00	3.00	7.00	6.00
13	4	2	0	2	1	2	1.25	1.00	6.00	1.40	1.00	1.00	1.00	2.00	1.00	1.00	1.00	6.00	5.00	7.00	2.00	1.00	2.00	1.00	1.00
14	1	4	1	3	2	3	5.75	3.33	4.67	5.80	5.00	7.00	6.00	5.00	3.00	3.00	4.00	5.00	5.00	4.00	6.00	6.00	6.00	6.00	5.00
15	5	4	0	5	5	5	6.00	7.00	5.33	6.60	6.00	5.00	6.00	7.00	7.00	7.00	7.00	5.00	6.00	5.00	7.00	6.00	6.00	7.00	7.00
16	4	4	0	4	2	4	3.00	2.00	1.00	4.40	5.00	3.00	1.00	3.00	3.00	2.00	1.00	1.00	1.00	1.00	6.00	4.00	5.00	4.00	3.00
17	4	1	0	3	4	1	1.25	1.00	6.33	3.20	1.00	1.00	2.00	1.00	1.00	1.00	1.00	5.00	7.00	7.00	3.00	4.00	3.00	4.00	2.00
18	2	4	0	1	2	3	3.00	5.33	5.33	2.80	2.00	3.00	3.00	4.00	6.00	5.00	5.00	7.00	5.00	4.00	1.00	5.00	4.00	3.00	1.00
19	2	1	1	5	2	5	4.25	5.67	6.33	2.20	4.00	3.00	5.00	5.00	7.00	5.00	5.00	6.00	6.00	7.00	2.00	2.00	3.00	2.00	2.00
20	1	2	0	2	3	1	1.25	3.67	4.67	5.80	1.00	1.00	2.00	1.00	3.00	5.00	3.00	6.00	4.00	4.00	6.00	5.00	7.00	6.00	5.00
21	3	3	1	1	5	5	4.75	2.33	5.67	5.80	6.00	6.00	3.00	4.00	3.00	3.00	1.00	5.00	6.00	6.00	6.00	6.00	5.00	6.00	6.00
22	3	2	0	3	4	5	5.75	6.33	6.00	3.40	5.00	6.00	6.00	6.00	7.00	5.00	7.00	7.00	6.00	5.00	5.00	2.00	2.00	4.00	4.00
23	3	4	1	1	4	2	2.75	5.67	6.00	3.00	1.00	4.00	3.00	3.00	6.00	5.00	6.00	6.00	7.00	5.00	4.00	2.00	2.00	3.00	4.00
24	3	5	1	1	3	4	5.50	5.67	1.67	5.80	4.00	6.00	7.00	5.00	6.00	5.00	6.00	1.00	2.00	2.00	6.00	6.00	6.00	6.00	5.00

25	5	1	0	4	5	2	1.00	4.67	1.67	3.40	1.00	1.00	1.00	1.00	6.00	4.00	4.00	2.00	1.00	2.00	2.00	3.00	4.00	4.00	4.00
26	5	2	1	2	2	2	4.75	5.67	4.00	5.00	4.00	3.00	7.00	5.00	6.00	6.00	5.00	4.00	4.00	4.00	6.00	4.00	5.00	4.00	6.00
27	4	2	0	3	1	3	4.25	1.67	3.33	3.20	2.00	5.00	6.00	4.00	1.00	2.00	2.00	2.00	5.00	3.00	2.00	4.00	2.00	3.00	5.00
28	1	2	0	1	1	2	2.50	5.67	5.33	5.40	2.00	6.00	1.00	1.00	5.00	6.00	6.00	5.00	5.00	6.00	5.00	5.00	6.00	6.00	5.00
29	4	2	1	4	1	4	4.75	6.00	2.67	3.80	4.00	7.00	3.00	5.00	6.00	5.00	7.00	2.00	3.00	3.00	6.00	3.00	1.00	4.00	5.00
30	4	3	0	1	1	1	4.00	1.67	1.33	2.40	4.00	2.00	6.00	4.00	1.00	2.00	2.00	1.00	2.00	1.00	3.00	2.00	3.00	2.00	2.00
31	4	1	0	5	2	5	3.75	3.67	6.00	5.40	3.00	3.00	3.00	6.00	3.00	5.00	3.00	7.00	6.00	5.00	5.00	5.00	5.00	5.00	7.00
32	3	1	0	2	2	3	4.00	4.33	2.00	5.60	6.00	3.00	3.00	4.00	4.00	6.00	3.00	2.00	2.00	2.00	6.00	5.00	3.00	7.00	7.00
33	4	5	1	2	3	4	4.75	4.67	1.00	2.80	6.00	2.00	5.00	6.00	4.00	6.00	4.00	1.00	1.00	1.00	2.00	3.00	4.00	3.00	2.00
34	4	4	0	5	1	4	2.75	5.33	1.67	5.80	1.00	3.00	4.00	3.00	5.00	5.00	6.00	2.00	1.00	2.00	6.00	6.00	5.00	7.00	5.00
35	3	3	0	3	1	5	2.25	2.00	3.00	4.00	1.00	1.00	1.00	6.00	2.00	2.00	2.00	4.00	4.00	1.00	4.00	4.00	5.00	4.00	3.00
36	2	2	0	2	5	3	4.25	2.33	5.00	3.40	3.00	6.00	4.00	4.00	2.00	3.00	2.00	6.00	6.00	3.00	4.00	4.00	3.00	4.00	2.00
37	1	2	0	3	1	5	6.00	4.33	4.00	4.40	6.00	5.00	6.00	7.00	3.00	4.00	6.00	4.00	5.00	3.00	5.00	4.00	3.00	5.00	5.00
38	1	1	1	5	4	4	2.25	5.33	5.33	6.00	3.00	2.00	2.00	2.00	5.00	6.00	5.00	5.00	6.00	5.00	7.00	7.00	7.00	5.00	4.00
39	1	1	0	1	2	2	1.25	1.67	4.67	1.20	1.00	1.00	2.00	1.00	3.00	1.00	1.00	5.00	5.00	4.00	1.00	1.00	2.00	1.00	1.00
40	5	3	0	4	5	5	3.50	3.67	2.67	5.00	2.00	5.00	4.00	3.00	3.00	4.00	4.00	3.00	3.00	2.00	6.00	5.00	3.00	6.00	5.00
41	3	4	0	3	1	2	5.50	1.67	1.67	2.40	5.00	5.00	6.00	6.00	2.00	2.00	1.00	2.00	1.00	2.00	2.00	2.00	3.00	2.00	3.00
42	2	2	0	1	2	2	1.00	1.67	5.67	1.20	1.00	1.00	1.00	1.00	2.00	1.00	2.00	6.00	5.00	6.00	1.00	1.00	2.00	1.00	1.00
43	5	4	0	4	4	2	6.75	5.33	3.33	4.40	7.00	7.00	6.00	7.00	5.00	6.00	5.00	2.00	4.00	4.00	4.00	3.00	4.00	5.00	6.00
44	2	2	1	4	1	3	2.25	6.33	2.67	4.40	1.00	2.00	1.00	5.00	5.00	7.00	7.00	3.00	2.00	3.00	5.00	4.00	6.00	4.00	3.00
45	5	4	0	2	1	4	5.50	4.67	4.00	3.40	3.00	7.00	5.00	7.00	5.00	4.00	5.00	5.00	4.00	3.00	2.00	4.00	4.00	4.00	3.00
46	5	2	1	5	3	3	6.25	5.67	1.67	3.00	6.00	7.00	5.00	7.00	5.00	6.00	6.00	2.00	2.00	1.00	2.00	2.00	3.00	3.00	5.00
47	3	5	1	4	1	3	5.75	5.67	2.00	5.80	6.00	6.00	5.00	6.00	7.00	4.00	6.00	3.00	1.00	2.00	5.00	6.00	6.00	6.00	6.00
48	2	4	0	3	3	5	4.00	6.67	5.67	5.80	2.00	4.00	6.00	4.00	6.00	7.00	7.00	7.00	5.00	5.00	6.00	6.00	6.00	6.00	5.00
49	5	5	0	1	3	4	2.25	6.33	2.00	4.40	2.00	2.00	3.00	2.00	5.00	7.00	7.00	1.00	2.00	3.00	4.00	3.00	5.00	4.00	6.00

50	2	2	0	3	3	1	5.25	4.67	1.33	4.20	6.00	7.00	3.00	5.00	3.00	5.00	6.00	1.00	2.00	1.00	5.00	3.00	4.00	4.00	5.00
51	1	1	0	2	2	1	1.50	1.67	5.33	1.20	1.00	2.00	2.00	1.00	2.00	1.00	2.00	6.00	5.00	5.00	1.00	1.00	1.00	1.00	2.00
52	1	5	1	5	2	1	5.75	2.67	1.33	5.60	5.00	6.00	6.00	6.00	3.00	2.00	3.00	1.00	1.00	2.00	5.00	6.00	5.00	6.00	6.00
53	2	2	0	1	3	3	4.00	3.67	6.67	5.60	2.00	5.00	5.00	4.00	4.00	3.00	4.00	7.00	6.00	7.00	5.00	6.00	6.00	6.00	5.00
54	1	4	1	1	2	4	5.00	5.33	5.00	4.80	2.00	6.00	6.00	6.00	5.00	5.00	6.00	5.00	5.00	5.00	4.00	3.00	5.00	5.00	7.00
55	1	1	0	2	1	2	2.25	1.00	6.67	1.20	2.00	2.00	3.00	2.00	1.00	1.00	1.00	6.00	7.00	7.00	1.00	1.00	1.00	1.00	2.00
56	3	2	0	3	1	2	2.50	1.33	5.33	1.80	3.00	2.00	3.00	2.00	1.00	1.00	2.00	7.00	6.00	3.00	2.00	2.00	3.00	1.00	1.00
57	4	5	0	4	2	5	4.75	2.00	1.67	2.40	6.00	3.00	5.00	5.00	1.00	2.00	3.00	2.00	1.00	2.00	2.00	3.00	3.00	2.00	2.00
58	3	4	0	3	4	5	4.50	3.67	4.00	5.40	5.00	5.00	5.00	3.00	3.00	5.00	3.00	5.00	5.00	2.00	7.00	6.00	5.00	5.00	4.00
59	3	5	1	3	2	2	5.75	6.33	5.67	2.40	4.00	7.00	7.00	5.00	7.00	7.00	5.00	7.00	5.00	5.00	3.00	2.00	2.00	2.00	3.00
60	2	5	1	2	1	1	2.50	2.00	1.33	5.20	2.00	1.00	1.00	6.00	2.00	2.00	2.00	2.00	1.00	1.00	4.00	7.00	7.00	5.00	3.00
61	5	4	0	2	3	4	1.25	3.67	5.00	4.40	1.00	2.00	1.00	1.00	4.00	4.00	3.00	5.00	5.00	5.00	5.00	5.00	5.00	3.00	4.00
62	2	5	1	4	3	3	1.25	6.67	1.33	3.60	1.00	2.00	1.00	1.00	6.00	7.00	7.00	1.00	2.00	1.00	5.00	2.00	3.00	4.00	4.00
63	1	2	0	1	1	1	1.25	1.33	5.33	2.00	2.00	1.00	1.00	1.00	2.00	1.00	1.00	5.00	6.00	5.00	2.00	3.00	1.00	2.00	2.00
64	2	3	1	3	1	4	4.00	6.67	6.67	5.80	5.00	5.00	3.00	3.00	6.00	7.00	7.00	7.00	7.00	6.00	7.00	6.00	6.00	5.00	5.00
65	4	4	0	4	3	5	5.75	5.33	5.00	6.20	7.00	4.00	7.00	5.00	5.00	5.00	6.00	6.00	5.00	4.00	6.00	7.00	5.00	7.00	6.00
66	5	3	0	3	1	1	1.50	1.00	6.33	1.60	1.00	2.00	2.00	1.00	1.00	1.00	1.00	6.00	6.00	7.00	1.00	2.00	3.00	1.00	1.00
67	1	4	1	2	4	3	3.00	5.33	5.33	2.60	2.00	2.00	4.00	4.00	5.00	5.00	6.00	5.00	5.00	6.00	3.00	2.00	3.00	2.00	3.00
68	1	2	0	4	3	4	5.50	2.00	2.33	2.40	5.00	5.00	6.00	6.00	1.00	3.00	2.00	2.00	2.00	3.00	4.00	1.00	2.00	3.00	2.00
69	2	3	0	1	2	1	4.75	1.67	1.00	1.60	5.00	4.00	6.00	4.00	2.00	1.00	2.00	1.00	1.00	1.00	1.00	1.00	1.00	3.00	2.00
70	5	4	1	5	3	5	4.50	5.67	7.00	5.80	2.00	6.00	6.00	4.00	5.00	7.00	5.00	7.00	7.00	7.00	6.00	6.00	6.00	6.00	5.00
71	1	1	0	3	5	2	3.75	4.00	6.00	5.60	5.00	3.00	3.00	4.00	2.00	5.00	5.00	6.00	7.00	5.00	5.00	5.00	7.00	6.00	5.00
72	1	3	0	5	2	2	2.25	5.67	5.67	5.60	4.00	1.00	1.00	3.00	5.00	6.00	6.00	5.00	6.00	6.00	6.00	5.00	3.00	7.00	7.00
73	1	1	0	5	1	2	2.75	4.33	3.33	3.80	3.00	3.00	3.00	2.00	4.00	6.00	3.00	3.00	2.00	5.00	2.00	5.00	6.00	3.00	3.00
74	1	5	1	5	5	4	5.75	5.33	2.33	5.20	6.00	5.00	6.00	6.00	5.00	5.00	6.00	3.00	2.00	2.00	5.00	6.00	5.00	4.00	6.00

75	3	5	0	2	5	5	6.75	6.67	3.67	5.40	7.00	7.00	6.00	7.00	6.00	7.00	7.00	4.00	3.00	4.00	6.00	6.00	5.00	5.00	5.00
76	4	3	0	4	1	2	2.50	1.33	5.67	2.00	2.00	3.00	3.00	2.00	1.00	2.00	1.00	5.00	7.00	5.00	2.00	2.00	2.00	2.00	2.00
77	1	5	0	2	5	5	5.50	5.67	1.33	5.60	6.00	5.00	5.00	6.00	5.00	6.00	6.00	2.00	1.00	1.00	6.00	5.00	6.00	6.00	5.00
78	2	1	0	2	4	2	1.25	1.33	5.00	5.40	1.00	2.00	1.00	1.00	1.00	1.00	2.00	6.00	6.00	3.00	5.00	6.00	5.00	6.00	5.00
79	4	4	0	1	5	2	1.25	3.67	1.00	5.20	1.00	2.00	1.00	1.00	3.00	3.00	5.00	1.00	1.00	1.00	7.00	5.00	6.00	5.00	3.00
80	2	4	1	1	5	5	2.50	6.67	3.67	5.80	3.00	3.00	2.00	2.00	7.00	7.00	6.00	3.00	3.00	5.00	6.00	6.00	5.00	6.00	6.00
81	1	3	0	2	3	4	3.00	3.33	6.67	5.00	5.00	1.00	3.00	3.00	4.00	2.00	4.00	6.00	7.00	7.00	5.00	5.00	6.00	5.00	4.00
82	2	5	0	1	1	3	3.25	2.67	1.33	5.80	5.00	2.00	3.00	3.00	3.00	3.00	2.00	1.00	2.00	1.00	6.00	6.00	6.00	6.00	5.00
83	4	1	0	2	4	1	3.25	2.67	2.33	1.80	2.00	2.00	3.00	6.00	4.00	3.00	1.00	2.00	2.00	3.00	2.00	2.00	2.00	1.00	2.00
84	2	5	0	1	2	1	2.25	5.67	3.00	2.20	2.00	2.00	3.00	2.00	6.00	5.00	6.00	3.00	4.00	2.00	2.00	2.00	2.00	2.00	3.00
85	3	5	0	5	4	2	4.25	1.00	1.33	2.00	6.00	3.00	4.00	4.00	1.00	1.00	1.00	1.00	1.00	2.00	2.00	3.00	1.00	2.00	2.00
86	1	3	1	5	3	5	6.00	5.33	5.67	5.80	5.00	6.00	6.00	7.00	5.00	5.00	6.00	6.00	6.00	5.00	5.00	5.00	6.00	7.00	6.00
87	1	2	0	2	3	3	1.75	1.33	5.33	2.00	3.00	1.00	1.00	2.00	2.00	1.00	1.00	5.00	5.00	6.00	2.00	2.00	3.00	1.00	2.00
88	5	1	0	2	2	2	1.75	1.00	7.00	3.00	1.00	2.00	2.00	2.00	1.00	1.00	1.00	7.00	7.00	7.00	4.00	3.00	3.00	3.00	2.00
89	1	1	0	1	2	1	1.75	1.00	6.33	1.40	2.00	2.00	2.00	1.00	1.00	1.00	1.00	7.00	6.00	6.00	1.00	2.00	2.00	1.00	1.00
90	3	1	1	1	2	5	6.75	1.00	1.33	2.20	6.00	7.00	7.00	7.00	1.00	1.00	1.00	1.00	2.00	1.00	3.00	2.00	2.00	2.00	2.00
91	1	4	1	4	5	3	4.50	5.33	2.00	5.40	6.00	2.00	6.00	4.00	5.00	6.00	5.00	2.00	2.00	2.00	5.00	5.00	5.00	6.00	6.00
92	2	5	0	5	4	1	1.50	3.33	5.67	4.20	1.00	2.00	2.00	1.00	4.00	1.00	5.00	6.00	6.00	5.00	5.00	4.00	6.00	4.00	2.00
93	3	4	0	2	5	3	6.00	2.33	5.67	5.80	7.00	5.00	5.00	7.00	3.00	2.00	2.00	6.00	6.00	5.00	5.00	6.00	6.00	6.00	6.00
94	2	4	0	3	3	3	5.75	7.00	5.00	5.60	7.00	7.00	4.00	5.00	7.00	7.00	7.00	7.00	3.00	5.00	5.00	5.00	5.00	7.00	6.00
95	2	5	1	3	4	5	6.00	6.67	4.33	6.60	7.00	6.00	7.00	4.00	7.00	6.00	7.00	3.00	5.00	5.00	7.00	6.00	6.00	7.00	7.00
96	3	4	0	1	3	3	2.50	1.67	4.33	5.20	3.00	3.00	1.00	3.00	2.00	1.00	2.00	3.00	5.00	5.00	7.00	4.00	4.00	5.00	6.00
97	2	3	1	3	1	4	5.50	1.67	5.33	2.00	5.00	6.00	5.00	6.00	2.00	2.00	1.00	6.00	5.00	5.00	1.00	3.00	1.00	3.00	2.00
98	1	5	0	5	3	2	5.75	5.00	5.67	6.20	6.00	5.00	6.00	6.00	5.00	5.00	5.00	5.00	7.00	5.00	5.00	6.00	6.00	7.00	7.00
99	1	1	0	2	4	3	4.00	2.00	7.00	2.60	4.00	5.00	4.00	3.00	2.00	2.00	2.00	7.00	7.00	7.00	4.00	2.00	2.00	3.00	2.00

100	3	2	0	1	2	1	1.00	1.33	5.67	1.20	1.00	1.00	1.00	1.00	1.00	1.00	2.00	5.00	6.00	6.00	2.00	1.00	1.00	1.00	1.00
101	3	3	0	5	5	1	3.25	2.00	3.00	4.80	4.00	3.00	3.00	3.00	2.00	3.00	1.00	2.00	3.00	4.00	3.00	4.00	6.00	5.00	6.00
102	3	4	1	1	3	3	1.25	5.67	6.00	4.60	1.00	2.00	1.00	1.00	7.00	5.00	5.00	7.00	6.00	5.00	6.00	3.00	4.00	4.00	6.00
103	4	3	1	4	5	4	6.00	6.67	6.00	6.20	6.00	7.00	6.00	5.00	6.00	7.00	7.00	6.00	6.00	6.00	7.00	6.00	6.00	7.00	5.00
104	1	5	0	1	1	4	7.00	3.67	6.33	5.60	7.00	7.00	7.00	7.00	4.00	3.00	4.00	7.00	7.00	5.00	5.00	6.00	5.00	6.00	6.00
105	2	4	0	2	1	2	5.75	5.67	4.67	2.60	6.00	7.00	5.00	5.00	6.00	6.00	5.00	5.00	4.00	5.00	2.00	3.00	4.00	2.00	2.00
106	1	1	1	4	1	4	4.75	3.67	2.33	5.40	5.00	5.00	5.00	4.00	2.00	3.00	6.00	2.00	3.00	2.00	3.00	7.00	5.00	5.00	7.00
107	4	2	0	2	1	1	1.25	1.00	6.33	5.60	2.00	1.00	1.00	1.00	1.00	1.00	1.00	6.00	6.00	7.00	6.00	5.00	5.00	7.00	5.00
108	4	2	0	4	5	3	5.00	5.67	3.33	4.00	6.00	5.00	5.00	4.00	6.00	6.00	5.00	4.00	2.00	4.00	1.00	5.00	7.00	3.00	4.00
109	4	2	0	1	3	3	2.75	1.33	2.00	1.80	3.00	3.00	3.00	2.00	1.00	1.00	2.00	3.00	1.00	2.00	2.00	2.00	3.00	1.00	1.00
110	1	2	0	4	2	4	5.00	3.00	5.00	5.40	6.00	2.00	6.00	6.00	3.00	4.00	2.00	5.00	6.00	4.00	5.00	5.00	6.00	6.00	5.00
111	3	2	0	1	4	5	3.25	2.00	5.33	2.20	3.00	3.00	3.00	4.00	2.00	2.00	2.00	4.00	6.00	6.00	2.00	2.00	2.00	2.00	3.00
112	3	5	1	2	2	2	6.00	4.67	4.33	5.80	7.00	5.00	5.00	7.00	5.00	3.00	6.00	3.00	4.00	6.00	6.00	6.00	6.00	6.00	5.00
113	1	1	1	2	2	2	5.50	4.33	4.67	4.20	5.00	6.00	5.00	6.00	4.00	6.00	3.00	3.00	5.00	6.00	6.00	4.00	3.00	4.00	4.00
114	2	1	0	4	4	3	5.50	1.67	5.00	2.40	6.00	5.00	5.00	6.00	2.00	1.00	2.00	5.00	5.00	5.00	3.00	3.00	1.00	2.00	3.00
115	3	1	0	4	3	2	1.75	1.67	5.67	5.60	2.00	2.00	2.00	1.00	2.00	1.00	2.00	6.00	6.00	5.00	6.00	5.00	3.00	7.00	7.00
116	3	2	1	1	3	1	2.75	1.67	2.00	1.20	2.00	3.00	2.00	4.00	2.00	1.00	2.00	2.00	2.00	2.00	1.00	1.00	2.00	1.00	1.00
117	1	4	0	1	2	5	4.25	1.00	1.33	2.00	3.00	4.00	6.00	4.00	1.00	1.00	1.00	2.00	1.00	1.00	2.00	2.00	2.00	2.00	2.00
118	5	5	1	5	4	4	2.00	5.67	5.33	6.00	2.00	2.00	2.00	2.00	6.00	6.00	5.00	5.00	5.00	6.00	6.00	6.00	5.00	7.00	6.00
119	2	5	0	4	5	5	6.00	7.00	5.00	6.40	5.00	6.00	6.00	7.00	7.00	7.00	7.00	5.00	5.00	5.00	7.00	6.00	6.00	7.00	6.00
120	1	2	0	3	1	2	1.00	2.33	1.67	1.60	1.00	1.00	1.00	1.00	2.00	2.00	3.00	1.00	2.00	2.00	1.00	2.00	3.00	1.00	1.00
121	4	5	1	5	5	4	7.00	7.00	5.67	6.80	7.00	7.00	7.00	7.00	7.00	7.00	7.00	5.00	6.00	6.00	7.00	7.00	7.00	7.00	6.00
122	1	3	1	3	2	3	5.50	6.00	4.00	4.00	5.00	5.00	5.00	7.00	7.00	5.00	6.00	2.00	4.00	6.00	5.00	4.00	3.00	3.00	5.00
123	4	1	0	3	3	5	3.00	4.33	2.33	2.40	2.00	2.00	4.00	4.00	4.00	5.00	4.00	1.00	2.00	4.00	2.00	2.00	3.00	3.00	2.00
124	3	2	0	3	1	5	3.00	1.67	2.33	1.80	3.00	4.00	2.00	3.00	2.00	2.00	1.00	1.00	3.00	3.00	1.00	2.00	3.00	1.00	2.00

125	5	5	1	5	5	4	5.50	6.00	2.33	6.60	7.00	5.00	5.00	5.00	7.00	6.00	5.00	1.00	3.00	3.00	5.00	7.00	7.00	7.00	7.00
126	2	4	0	1	2	1	2.75	5.00	1.67	3.00	3.00	3.00	3.00	2.00	5.00	5.00	5.00	1.00	2.00	2.00	3.00	3.00	5.00	2.00	2.00
127	1	1	0	4	3	1	6.75	5.00	4.00	5.60	7.00	6.00	7.00	7.00	6.00	5.00	4.00	5.00	4.00	3.00	5.00	6.00	5.00	6.00	6.00
128	3	1	0	3	5	1	4.25	4.00	3.33	3.80	3.00	4.00	5.00	5.00	4.00	5.00	3.00	5.00	2.00	3.00	5.00	2.00	2.00	4.00	6.00
129	5	2	1	3	3	1	2.25	1.33	5.33	2.60	2.00	3.00	2.00	2.00	2.00	1.00	1.00	6.00	6.00	4.00	2.00	3.00	3.00	2.00	3.00
130	1	2	1	2	5	1	2.50	1.67	1.67	5.40	3.00	2.00	2.00	3.00	1.00	2.00	2.00	2.00	2.00	1.00	6.00	6.00	6.00	5.00	4.00
131	1	2	0	1	4	3	1.50	5.33	5.67	2.20	2.00	1.00	2.00	1.00	3.00	6.00	7.00	6.00	6.00	5.00	2.00	2.00	2.00	2.00	3.00
132	4	4	0	4	2	5	2.75	2.33	6.33	3.00	3.00	3.00	3.00	2.00	2.00	3.00	2.00	5.00	7.00	7.00	3.00	3.00	5.00	2.00	2.00
133	5	4	1	4	4	4	6.25	5.67	3.33	5.60	6.00	6.00	6.00	7.00	6.00	5.00	6.00	6.00	2.00	2.00	5.00	6.00	6.00	6.00	5.00
134	5	4	1	2	3	5	5.50	2.67	6.33	3.80	5.00	5.00	6.00	6.00	3.00	3.00	2.00	6.00	7.00	6.00	6.00	2.00	3.00	4.00	4.00
135	5	4	1	3	2	3	4.50	3.67	3.00	4.60	5.00	1.00	6.00	6.00	4.00	4.00	3.00	3.00	5.00	1.00	2.00	6.00	6.00	4.00	5.00
136	4	3	1	4	3	5	3.25	2.67	5.67	3.00	1.00	5.00	4.00	3.00	3.00	2.00	3.00	5.00	6.00	6.00	3.00	3.00	5.00	2.00	2.00
137	2	5	0	2	5	4	3.75	2.00	2.33	5.20	3.00	3.00	4.00	5.00	2.00	2.00	2.00	3.00	2.00	2.00	5.00	6.00	5.00	5.00	5.00
138	2	1	0	1	3	5	1.50	2.00	6.00	3.80	2.00	1.00	2.00	1.00	2.00	2.00	2.00	6.00	6.00	6.00	4.00	4.00	6.00	3.00	2.00
139	5	4	0	4	2	5	4.25	6.00	6.00	6.00	5.00	6.00	2.00	4.00	5.00	7.00	6.00	6.00	6.00	6.00	6.00	5.00	7.00	7.00	5.00
140	2	2	0	5	3	1	4.50	3.00	3.33	3.80	1.00	5.00	6.00	6.00	4.00	2.00	3.00	2.00	3.00	5.00	4.00	4.00	6.00	3.00	2.00
141	1	2	0	1	1	5	1.25	1.33	5.00	1.00	1.00	2.00	1.00	1.00	1.00	1.00	2.00	5.00	5.00	5.00	1.00	1.00	1.00	1.00	1.00
142	3	2	0	2	2	2	6.25	5.67	5.67	2.60	7.00	6.00	7.00	5.00	5.00	7.00	5.00	6.00	6.00	5.00	3.00	2.00	3.00	2.00	3.00
143	2	2	0	5	5	4	2.50	3.33	5.33	3.00	3.00	2.00	3.00	2.00	5.00	3.00	2.00	5.00	6.00	5.00	3.00	3.00	5.00	2.00	2.00
144	1	2	0	1	1	5	4.75	1.67	1.67	5.00	5.00	6.00	4.00	4.00	2.00	2.00	1.00	2.00	1.00	2.00	6.00	5.00	3.00	6.00	5.00
145	1	4	0	4	2	1	6.50	3.00	2.67	5.60	5.00	7.00	7.00	7.00	2.00	2.00	5.00	2.00	3.00	3.00	5.00	6.00	6.00	6.00	5.00
146	1	5	0	4	4	1	5.25	1.67	6.33	3.60	4.00	7.00	5.00	5.00	2.00	1.00	2.00	6.00	6.00	7.00	6.00	2.00	2.00	4.00	4.00
147	3	4	0	5	1	5	5.00	5.33	6.67	3.60	7.00	3.00	5.00	5.00	6.00	5.00	5.00	6.00	7.00	7.00	5.00	5.00	4.00	3.00	1.00
148	3	1	0	3	2	1	3.00	2.67	2.33	1.60	2.00	3.00	5.00	2.00	3.00	3.00	2.00	2.00	3.00	2.00	2.00	1.00	2.00	1.00	2.00
149	2	2	0	2	2	1	1.25	4.67	2.67	2.20	1.00	1.00	2.00	1.00	6.00	5.00	3.00	3.00	2.00	3.00	2.00	2.00	2.00	2.00	3.00

150	4	4	1	5	5	4	5.50	6.67	6.67	6.40	7.00	7.00	4.00	4.00	7.00	6.00	7.00	7.00	6.00	7.00	5.00	7.00	7.00	7.00	6.00
151	2	3	0	1	4	5	3.50	4.33	2.33	3.00	2.00	5.00	3.00	4.00	2.00	5.00	6.00	2.00	2.00	3.00	3.00	3.00	5.00	3.00	1.00
152	1	5	1	2	5	2	4.25	2.33	1.33	2.20	3.00	6.00	4.00	4.00	3.00	1.00	3.00	1.00	2.00	1.00	2.00	2.00	3.00	2.00	2.00
153	4	2	0	2	2	2	2.50	5.67	5.00	2.40	2.00	3.00	3.00	2.00	5.00	7.00	5.00	5.00	4.00	6.00	3.00	2.00	2.00	2.00	3.00
154	1	4	1	5	5	5	6.25	7.00	5.67	6.00	6.00	7.00	7.00	5.00	7.00	7.00	7.00	6.00	5.00	6.00	5.00	5.00	6.00	7.00	7.00
155	5	2	0	2	3	3	2.25	5.00	1.67	2.60	2.00	3.00	2.00	2.00	7.00	3.00	5.00	2.00	2.00	1.00	2.00	2.00	3.00	3.00	3.00
156	4	3	1	4	2	3	2.50	2.67	5.00	2.00	1.00	2.00	4.00	3.00	2.00	3.00	3.00	5.00	5.00	5.00	2.00	2.00	2.00	2.00	2.00
157	2	4	1	1	3	3	6.25	2.00	5.00	2.40	7.00	6.00	5.00	7.00	2.00	3.00	1.00	5.00	6.00	4.00	3.00	2.00	3.00	2.00	2.00
158	1	4	1	2	1	1	1.00	2.00	3.67	1.40	1.00	1.00	1.00	1.00	2.00	2.00	2.00	5.00	4.00	2.00	1.00	1.00	2.00	1.00	2.00
159	2	3	0	1	2	3	4.25	4.67	4.00	5.40	5.00	4.00	4.00	4.00	6.00	4.00	4.00	4.00	4.00	4.00	6.00	5.00	4.00	6.00	6.00
160	3	4	0	2	4	4	6.25	6.67	5.67	6.00	6.00	5.00	7.00	7.00	7.00	7.00	6.00	5.00	5.00	7.00	5.00	6.00	6.00	7.00	6.00
161	2	1	0	2	2	4	3.75	3.00	6.00	2.20	5.00	4.00	3.00	3.00	4.00	2.00	3.00	5.00	6.00	7.00	3.00	2.00	2.00	2.00	2.00
162	2	1	0	3	2	1	1.25	1.00	7.00	1.40	1.00	1.00	1.00	2.00	1.00	1.00	1.00	7.00	7.00	7.00	1.00	1.00	2.00	1.00	2.00
163	1	5	0	3	2	2	3.75	6.00	3.33	2.60	2.00	5.00	5.00	3.00	6.00	7.00	5.00	2.00	2.00	6.00	2.00	3.00	3.00	2.00	3.00
164	2	2	0	3	1	1	1.25	1.33	1.67	1.20	1.00	2.00	1.00	1.00	1.00	1.00	2.00	2.00	2.00	1.00	1.00	1.00	2.00	1.00	1.00
165	4	2	0	4	3	5	4.75	2.00	3.67	5.20	3.00	6.00	5.00	5.00	2.00	2.00	2.00	3.00	5.00	3.00	7.00	6.00	5.00	5.00	3.00
166	3	5	1	5	3	5	6.25	7.00	5.00	3.80	5.00	6.00	7.00	7.00	7.00	7.00	7.00	5.00	5.00	5.00	3.00	4.00	4.00	5.00	3.00
167	2	1	1	1	3	5	1.50	5.00	5.67	3.80	1.00	2.00	2.00	1.00	5.00	5.00	5.00	5.00	7.00	5.00	5.00	3.00	4.00	4.00	3.00
168	1	5	1	5	3	1	4.25	6.00	2.00	4.20	3.00	7.00	4.00	3.00	5.00	7.00	6.00	2.00	2.00	2.00	3.00	3.00	5.00	4.00	6.00
169	3	4	1	4	5	2	4.25	3.67	2.67	4.80	3.00	3.00	6.00	5.00	4.00	3.00	4.00	3.00	3.00	2.00	6.00	4.00	2.00	5.00	7.00
170	3	1	0	1	1	2	1.75	2.33	3.00	1.20	2.00	2.00	2.00	1.00	1.00	3.00	3.00	3.00	3.00	3.00	1.00	1.00	2.00	1.00	1.00
171	3	2	1	5	3	5	6.75	6.33	6.00	6.40	7.00	6.00	7.00	7.00	7.00	5.00	7.00	5.00	7.00	6.00	7.00	7.00	6.00	7.00	5.00
172	2	3	0	2	2	4	3.75	5.00	5.33	5.60	4.00	4.00	3.00	4.00	4.00	6.00	5.00	6.00	5.00	5.00	6.00	5.00	5.00	5.00	7.00
173	3	2	0	4	5	1	6.25	5.00	4.33	5.20	5.00	6.00	7.00	7.00	5.00	5.00	5.00	5.00	5.00	3.00	6.00	5.00	4.00	6.00	5.00
174	2	4	0	2	1	2	3.00	2.33	4.67	5.80	3.00	3.00	3.00	3.00	2.00	3.00	2.00	5.00	5.00	4.00	6.00	6.00	5.00	7.00	5.00

175	3	4	0	5	4	3	6.00	3.33	4.33	4.00	7.00	5.00	5.00	7.00	4.00	2.00	4.00	6.00	2.00	5.00	4.00	4.00	5.00	3.00	4.00
176	4	1	1	4	3	1	1.75	1.00	6.33	2.60	2.00	3.00	1.00	1.00	1.00	1.00	1.00	5.00	7.00	7.00	3.00	2.00	2.00	3.00	3.00
177	2	1	0	4	1	4	1.25	3.67	1.33	4.60	1.00	1.00	2.00	1.00	3.00	3.00	5.00	1.00	1.00	2.00	6.00	4.00	3.00	4.00	6.00
178	5	3	0	2	4	4	4.25	1.67	5.67	2.60	6.00	5.00	2.00	4.00	2.00	2.00	1.00	6.00	6.00	5.00	2.00	3.00	1.00	4.00	3.00
179	1	4	1	4	1	4	5.25	3.67	5.33	3.40	7.00	5.00	4.00	5.00	3.00	3.00	5.00	4.00	7.00	5.00	2.00	2.00	3.00	4.00	6.00
180	1	5	0	5	4	3	2.25	4.00	3.67	5.00	2.00	3.00	2.00	2.00	3.00	3.00	6.00	3.00	4.00	4.00	7.00	4.00	3.00	5.00	6.00
181	2	3	0	2	5	4	3.75	3.33	6.00	3.20	3.00	2.00	5.00	5.00	4.00	2.00	4.00	6.00	6.00	6.00	2.00	3.00	5.00	3.00	3.00
182	3	4	0	1	2	4	5.75	3.33	5.00	4.20	6.00	6.00	6.00	5.00	4.00	3.00	3.00	6.00	4.00	5.00	4.00	6.00	6.00	2.00	3.00
183	1	1	0	1	2	4	1.50	5.67	3.00	5.20	1.00	2.00	2.00	1.00	4.00	6.00	7.00	3.00	4.00	2.00	6.00	5.00	3.00	6.00	6.00
184	4	5	1	4	5	3	6.25	5.00	3.00	3.60	7.00	6.00	6.00	6.00	4.00	7.00	4.00	1.00	5.00	3.00	3.00	5.00	3.00	3.00	4.00
185	4	1	0	1	4	5	6.00	2.67	6.00	4.00	7.00	7.00	5.00	5.00	3.00	3.00	2.00	5.00	6.00	7.00	4.00	3.00	4.00	6.00	3.00
186	1	1	1	1	3	4	1.00	1.00	6.00	1.00	1.00	1.00	1.00	1.00	1.00	1.00	1.00	7.00	5.00	6.00	1.00	1.00	1.00	1.00	1.00
187	2	2	0	4	1	5	5.25	6.67	5.33	2.20	7.00	4.00	6.00	4.00	7.00	7.00	6.00	4.00	7.00	5.00	3.00	2.00	3.00	1.00	2.00
188	2	3	0	2	1	2	2.75	1.33	2.00	1.60	2.00	3.00	3.00	3.00	1.00	1.00	2.00	2.00	2.00	2.00	1.00	1.00	2.00	2.00	2.00
189	2	2	0	1	2	2	1.00	3.00	5.67	1.40	1.00	1.00	1.00	1.00	3.00	3.00	3.00	5.00	6.00	6.00	1.00	1.00	2.00	1.00	2.00
190	4	1	0	3	1	3	1.50	5.67	4.00	5.00	1.00	1.00	2.00	2.00	5.00	5.00	7.00	5.00	2.00	5.00	5.00	7.00	6.00	4.00	3.00
191	1	3	0	1	2	1	3.75	1.33	1.33	2.00	3.00	2.00	5.00	5.00	1.00	2.00	1.00	1.00	2.00	1.00	1.00	2.00	3.00	2.00	2.00
192	2	1	1	1	3	4	5.50	1.67	3.33	6.00	6.00	6.00	5.00	5.00	1.00	2.00	2.00	4.00	4.00	2.00	7.00	7.00	5.00	6.00	5.00
193	1	3	0	4	5	4	5.25	3.33	2.67	4.00	6.00	5.00	5.00	5.00	4.00	2.00	4.00	3.00	3.00	2.00	4.00	3.00	4.00	3.00	6.00
194	4	1	0	3	5	4	2.75	3.67	2.33	3.40	2.00	3.00	3.00	3.00	4.00	4.00	3.00	3.00	2.00	2.00	3.00	5.00	4.00	2.00	3.00
195	4	2	0	2	5	4	2.50	5.00	2.67	4.60	2.00	2.00	3.00	3.00	5.00	5.00	5.00	2.00	3.00	3.00	4.00	4.00	3.00	6.00	6.00
196	3	3	1	5	4	4	6.25	6.67	6.33	6.00	6.00	7.00	5.00	7.00	6.00	7.00	7.00	7.00	5.00	7.00	7.00	6.00	5.00	5.00	7.00
197	3	2	0	5	1	4	5.75	5.67	5.67	4.00	5.00	7.00	5.00	6.00	6.00	6.00	5.00	6.00	6.00	5.00	4.00	6.00	5.00	2.00	3.00
198	4	4	0	3	1	1	5.00	2.00	5.67	2.40	4.00	5.00	6.00	5.00	2.00	3.00	1.00	5.00	6.00	6.00	2.00	3.00	1.00	3.00	3.00
199	3	2	0	2	1	4	6.25	4.00	2.33	3.20	7.00	6.00	6.00	6.00	5.00	4.00	3.00	2.00	2.00	3.00	2.00	3.00	5.00	3.00	3.00

200	2	3	0	5	5	2	4.00	2.67	5.00	5.20	4.00	5.00	5.00	2.00	3.00	3.00	2.00	5.00	5.00	5.00	6.00	5.00	3.00	6.00	6.00
201	1	1	1	5	3	3	3.25	4.00	5.00	5.80	3.00	1.00	4.00	5.00	3.00	5.00	4.00	6.00	4.00	5.00	6.00	5.00	7.00	6.00	5.00
202	3	5	1	2	3	3	6.75	6.33	5.67	6.00	6.00	7.00	7.00	7.00	5.00	7.00	7.00	6.00	6.00	5.00	6.00	6.00	6.00	6.00	6.00
203	5	1	0	4	3	1	1.75	1.00	4.67	1.80	1.00	3.00	2.00	1.00	1.00	1.00	1.00	4.00	6.00	4.00	1.00	2.00	3.00	2.00	1.00
204	3	4	0	4	1	1	5.50	5.33	3.67	5.80	6.00	5.00	6.00	5.00	6.00	6.00	4.00	3.00	4.00	4.00	6.00	5.00	7.00	5.00	6.00
205	2	1	1	2	2	4	4.00	4.67	2.67	4.00	4.00	3.00	4.00	5.00	6.00	5.00	3.00	3.00	3.00	2.00	4.00	4.00	2.00	6.00	4.00
206	1	1	1	5	1	5	4.50	1.33	5.33	2.20	5.00	3.00	4.00	6.00	1.00	2.00	1.00	6.00	5.00	5.00	3.00	1.00	1.00	1.00	5.00
207	4	5	0	2	1	2	5.75	2.33	2.67	3.60	6.00	5.00	6.00	6.00	2.00	1.00	4.00	2.00	3.00	3.00	4.00	3.00	2.00	5.00	4.00
208	3	5	0	2	2	4	5.75	2.33	5.33	5.60	6.00	6.00	5.00	6.00	1.00	3.00	3.00	6.00	5.00	5.00	6.00	6.00	5.00	5.00	6.00
209	2	1	0	3	3	5	2.50	4.33	2.33	2.20	3.00	3.00	1.00	3.00	3.00	5.00	5.00	3.00	2.00	2.00	3.00	1.00	1.00	2.00	4.00
210	5	3	0	3	2	3	1.75	1.67	5.00	1.60	1.00	2.00	2.00	2.00	1.00	3.00	1.00	6.00	6.00	3.00	1.00	1.00	2.00	2.00	2.00
211	1	1	0	3	3	3	2.00	5.67	4.67	2.20	2.00	2.00	2.00	2.00	7.00	3.00	7.00	5.00	5.00	4.00	2.00	3.00	1.00	2.00	3.00
212	1	4	0	3	3	1	4.00	5.33	4.67	6.00	3.00	4.00	5.00	4.00	6.00	5.00	5.00	5.00	4.00	5.00	6.00	6.00	6.00	6.00	6.00
213	2	5	0	5	3	4	2.50	1.67	3.33	4.00	2.00	3.00	3.00	2.00	2.00	2.00	1.00	3.00	2.00	5.00	4.00	4.00	2.00	5.00	5.00
214	1	1	0	2	3	3	3.00	1.33	3.33	3.40	4.00	2.00	3.00	3.00	2.00	1.00	1.00	4.00	4.00	2.00	4.00	4.00	3.00	4.00	2.00
215	1	3	1	3	5	5	6.00	6.33	5.67	6.40	7.00	5.00	7.00	5.00	6.00	6.00	7.00	5.00	7.00	5.00	7.00	7.00	6.00	6.00	6.00
216	4	1	1	1	3	4	6.50	6.00	5.33	3.00	7.00	5.00	7.00	7.00	5.00	6.00	7.00	6.00	4.00	6.00	3.00	2.00	2.00	4.00	4.00
217	3	2	0	5	4	1	2.50	1.33	3.67	4.40	3.00	1.00	4.00	2.00	1.00	1.00	2.00	3.00	3.00	5.00	5.00	3.00	5.00	3.00	6.00
218	1	1	1	1	1	3	4.00	1.33	2.67	1.40	3.00	4.00	5.00	4.00	1.00	2.00	1.00	3.00	2.00	3.00	1.00	1.00	1.00	2.00	2.00
219	4	4	1	2	4	5	6.75	6.67	6.67	6.80	7.00	6.00	7.00	7.00	6.00	7.00	7.00	6.00	7.00	7.00	7.00	7.00	7.00	6.00	7.00
220	5	1	1	3	3	2	6.75	4.67	2.33	3.20	7.00	7.00	6.00	7.00	5.00	6.00	3.00	2.00	3.00	2.00	4.00	4.00	2.00	2.00	4.00
221	1	2	0	2	1	1	4.25	5.33	3.67	5.80	4.00	5.00	2.00	6.00	6.00	5.00	5.00	2.00	4.00	5.00	6.00	6.00	6.00	6.00	5.00
222	2	3	0	5	2	2	2.75	4.00	6.00	2.60	2.00	3.00	3.00	3.00	3.00	5.00	4.00	5.00	7.00	6.00	3.00	1.00	1.00	4.00	4.00
223	1	1	0	2	1	1	1.00	1.00	5.33	1.00	1.00	1.00	1.00	1.00	1.00	1.00	1.00	4.00	6.00	6.00	1.00	1.00	1.00	1.00	1.00
224	2	2	0	2	1	4	6.25	1.33	3.00	3.80	7.00	7.00	6.00	5.00	1.00	1.00	2.00	4.00	2.00	3.00	3.00	4.00	6.00	2.00	4.00

225	4	3	0	1	4	4	1.00	1.00	5.00	2.60	1.00	1.00	1.00	1.00	1.00	1.00	1.00	4.00	4.00	7.00	3.00	1.00	2.00	4.00	3.00
226	3	4	1	5	3	3	6.25	7.00	5.33	6.20	7.00	7.00	4.00	7.00	7.00	7.00	7.00	5.00	5.00	6.00	7.00	7.00	7.00	5.00	5.00
227	3	3	1	1	5	4	6.00	7.00	5.33	2.80	7.00	5.00	6.00	6.00	7.00	7.00	7.00	5.00	5.00	6.00	3.00	2.00	3.00	1.00	5.00
228	4	4	1	5	5	4	6.25	5.67	3.67	6.20	6.00	6.00	7.00	6.00	5.00	6.00	6.00	4.00	4.00	3.00	7.00	7.00	5.00	5.00	7.00
229	2	3	1	5	2	5	4.00	5.67	6.00	5.80	3.00	4.00	4.00	5.00	5.00	5.00	7.00	7.00	5.00	6.00	6.00	5.00	6.00	6.00	6.00
230	5	4	0	3	2	3	6.75	5.33	1.67	4.40	7.00	7.00	6.00	7.00	6.00	4.00	6.00	1.00	2.00	2.00	5.00	4.00	5.00	5.00	3.00
231	3	4	0	2	3	1	2.25	5.67	3.67	2.40	2.00	2.00	2.00	3.00	6.00	5.00	6.00	5.00	3.00	3.00	2.00	3.00	2.00	2.00	3.00
232	2	2	0	3	1	5	1.25	1.33	3.00	1.00	1.00	1.00	1.00	2.00	1.00	2.00	1.00	4.00	2.00	3.00	1.00	1.00	1.00	1.00	1.00
233	2	1	0	1	2	1	3.50	2.00	2.33	5.80	3.00	5.00	2.00	4.00	2.00	2.00	2.00	2.00	2.00	3.00	6.00	6.00	5.00	6.00	6.00
234	1	4	1	3	5	5	6.75	7.00	5.00	6.20	7.00	7.00	6.00	7.00	7.00	7.00	7.00	4.00	4.00	7.00	7.00	5.00	6.00	7.00	6.00
235	4	5	1	4	4	5	6.00	5.67	5.00	6.40	7.00	7.00	5.00	5.00	6.00	5.00	6.00	5.00	5.00	5.00	7.00	6.00	5.00	7.00	7.00
236	4	2	0	3	2	1	1.25	1.33	7.00	1.60	2.00	1.00	1.00	1.00	2.00	1.00	1.00	7.00	7.00	7.00	1.00	1.00	2.00	2.00	2.00
237	1	5	1	4	3	2	7.00	5.00	2.67	6.00	7.00	7.00	7.00	7.00	5.00	5.00	5.00	3.00	3.00	2.00	7.00	6.00	5.00	6.00	6.00
238	1	1	1	4	1	4	3.00	2.33	2.00	5.60	3.00	5.00	1.00	3.00	2.00	2.00	3.00	2.00	2.00	2.00	5.00	6.00	6.00	5.00	6.00
239	1	1	0	1	5	3	2.50	5.33	5.67	3.80	2.00	3.00	2.00	3.00	6.00	6.00	4.00	6.00	6.00	5.00	5.00	5.00	3.00	3.00	3.00
240	5	1	0	5	1	1	6.50	6.00	2.67	3.40	7.00	7.00	7.00	5.00	6.00	6.00	6.00	3.00	3.00	2.00	4.00	3.00	5.00	2.00	3.00
241	4	2	1	2	1	3	5.00	1.33	3.67	1.80	4.00	6.00	6.00	4.00	1.00	2.00	1.00	2.00	5.00	4.00	1.00	2.00	3.00	1.00	2.00
242	3	4	0	1	2	3	5.75	5.33	1.00	2.40	7.00	5.00	6.00	5.00	5.00	6.00	5.00	1.00	1.00	1.00	4.00	3.00	1.00	2.00	2.00
243	5	4	1	4	4	4	6.25	6.67	5.67	6.60	5.00	7.00	6.00	7.00	7.00	7.00	6.00	5.00	7.00	5.00	7.00	7.00	7.00	7.00	5.00
244	5	3	0	3	5	4	5.50	5.33	5.67	5.60	7.00	5.00	5.00	5.00	5.00	6.00	5.00	5.00	6.00	6.00	7.00	5.00	5.00	5.00	6.00
245	1	2	0	3	4	1	4.75	3.33	5.33	5.80	5.00	3.00	7.00	4.00	3.00	4.00	3.00	6.00	6.00	4.00	5.00	7.00	7.00	4.00	6.00
246	3	5	1	3	4	3	2.50	6.67	1.33	6.00	2.00	3.00	3.00	2.00	7.00	6.00	7.00	1.00	2.00	1.00	7.00	6.00	5.00	6.00	6.00
247	2	5	1	3	3	5	6.50	7.00	6.67	6.00	5.00	7.00	7.00	7.00	7.00	7.00	7.00	6.00	7.00	7.00	6.00	6.00	6.00	6.00	6.00
248	3	4	0	1	3	5	5.50	6.67	5.00	3.80	4.00	6.00	6.00	6.00	6.00	7.00	7.00	4.00	6.00	5.00	4.00	4.00	2.00	6.00	3.00
249	2	2	0	5	3	3	4.50	5.33	5.33	2.60	4.00	6.00	2.00	6.00	6.00	5.00	5.00	5.00	6.00	5.00	3.00	2.00	3.00	3.00	2.00

250	2	5	1	4	2	5	6.00	6.67	4.33	6.60	5.00	7.00	6.00	6.00	7.00	7.00	6.00	5.00	5.00	3.00	7.00	7.00	7.00	7.00	5.00
251	3	2	0	2	3	3	1.50	1.00	5.00	4.80	2.00	1.00	2.00	1.00	1.00	1.00	1.00	6.00	3.00	6.00	4.00	4.00	5.00	6.00	5.00
252	5	3	0	5	1	5	3.25	5.33	2.67	5.60	3.00	3.00	4.00	3.00	6.00	5.00	5.00	3.00	2.00	3.00	6.00	5.00	5.00	6.00	6.00
253	3	4	1	2	5	4	5.25	5.67	4.67	5.20	5.00	5.00	5.00	6.00	5.00	6.00	6.00	3.00	6.00	5.00	7.00	5.00	3.00	5.00	6.00
254	4	5	0	3	2	5	5.75	2.33	4.33	5.20	6.00	5.00	6.00	6.00	3.00	2.00	2.00	3.00	7.00	3.00	5.00	4.00	3.00	7.00	7.00
255	2	5	0	3	4	5	4.25	3.67	3.00	2.80	4.00	4.00	4.00	5.00	5.00	1.00	5.00	3.00	3.00	3.00	2.00	3.00	5.00	2.00	2.00
256	2	1	0	1	4	5	2.50	5.67	3.67	2.20	3.00	3.00	1.00	3.00	6.00	5.00	6.00	4.00	4.00	3.00	2.00	3.00	1.00	3.00	2.00
257	4	1	0	1	3	5	5.50	3.67	2.67	4.40	6.00	5.00	6.00	5.00	4.00	5.00	2.00	2.00	3.00	3.00	4.00	3.00	5.00	5.00	5.00
258	4	2	0	3	1	4	2.50	2.67	5.00	2.60	2.00	2.00	3.00	3.00	3.00	2.00	3.00	5.00	6.00	4.00	2.00	2.00	3.00	3.00	3.00
259	2	3	0	4	4	2	2.00	4.33	2.33	5.60	2.00	2.00	2.00	2.00	5.00	3.00	5.00	2.00	2.00	3.00	5.00	6.00	6.00	6.00	5.00
260	5	3	0	1	5	2	6.00	1.33	6.67	4.40	6.00	6.00	6.00	6.00	2.00	1.00	1.00	7.00	7.00	6.00	3.00	4.00	6.00	5.00	4.00
261	3	5	0	5	3	3	3.00	2.00	2.33	3.60	3.00	3.00	4.00	2.00	2.00	2.00	2.00	3.00	2.00	2.00	4.00	4.00	2.00	5.00	3.00
262	3	1	0	1	1	1	1.00	1.67	4.67	1.20	1.00	1.00	1.00	1.00	2.00	1.00	2.00	6.00	7.00	1.00	1.00	1.00	2.00	1.00	1.00
263	1	3	1	2	2	5	2.50	2.33	2.67	3.40	3.00	1.00	4.00	2.00	2.00	3.00	2.00	3.00	3.00	2.00	4.00	3.00	4.00	2.00	4.00
264	2	5	1	4	3	3	2.25	3.33	6.67	5.60	2.00	2.00	2.00	3.00	3.00	2.00	5.00	7.00	7.00	6.00	7.00	5.00	4.00	5.00	7.00
265	1	4	0	3	1	2	5.25	4.33	2.67	2.80	5.00	6.00	6.00	4.00	5.00	6.00	2.00	3.00	2.00	3.00	3.00	2.00	3.00	2.00	4.00
266	1	2	1	5	4	5	1.50	3.00	5.33	5.80	1.00	2.00	1.00	2.00	4.00	3.00	2.00	5.00	7.00	4.00	7.00	6.00	6.00	5.00	5.00
267	2	1	0	3	5	3	5.25	5.00	2.00	3.60	6.00	5.00	5.00	5.00	3.00	7.00	5.00	2.00	2.00	2.00	3.00	3.00	3.00	5.00	4.00
268	1	3	0	2	2	5	5.75	6.00	6.00	4.40	6.00	5.00	6.00	6.00	5.00	7.00	6.00	5.00	7.00	6.00	4.00	6.00	5.00	4.00	3.00
269	5	3	1	5	4	5	6.75	6.00	6.67	6.40	7.00	6.00	7.00	7.00	6.00	5.00	7.00	7.00	6.00	7.00	5.00	7.00	7.00	6.00	7.00
270	3	5	0	2	3	2	4.00	4.67	1.67	5.60	4.00	4.00	2.00	6.00	5.00	5.00	4.00	1.00	2.00	2.00	6.00	5.00	5.00	6.00	6.00
271	3	4	0	3	2	3	2.75	5.67	4.67	3.00	4.00	2.00	3.00	2.00	6.00	6.00	5.00	6.00	3.00	5.00	2.00	3.00	5.00	3.00	2.00
272	3	3	0	2	3	5	4.75	6.33	6.67	5.80	5.00	5.00	3.00	6.00	7.00	6.00	6.00	7.00	6.00	7.00	6.00	6.00	6.00	5.00	6.00
273	2	1	0	1	1	5	5.00	5.67	1.00	3.60	5.00	3.00	7.00	5.00	5.00	5.00	7.00	1.00	1.00	1.00	4.00	3.00	3.00	3.00	5.00
274	5	3	1	4	5	3	1.25	2.00	7.00	2.60	1.00	1.00	1.00	2.00	2.00	2.00	2.00	7.00	7.00	7.00	3.00	1.00	2.00	5.00	2.00

275	2	1	0	2	3	4	4.00	3.33	3.33	3.60	4.00	3.00	5.00	4.00	3.00	5.00	2.00	3.00	2.00	5.00	3.00	4.00	3.00	5.00	3.00
276	1	3	1	1	3	4	3.50	5.67	2.00	5.80	3.00	3.00	3.00	5.00	5.00	6.00	6.00	2.00	2.00	2.00	7.00	5.00	5.00	7.00	5.00
277	4	1	0	3	2	3	2.00	3.00	7.00	3.20	2.00	2.00	2.00	2.00	3.00	3.00	3.00	7.00	7.00	7.00	3.00	5.00	4.00	1.00	3.00
278	2	3	0	2	5	4	6.50	6.00	5.00	5.60	6.00	7.00	7.00	6.00	6.00	6.00	6.00	5.00	5.00	5.00	7.00	5.00	3.00	6.00	7.00
279	2	4	0	2	4	5	3.50	5.67	1.33	2.40	4.00	4.00	4.00	2.00	6.00	6.00	5.00	1.00	2.00	1.00	2.00	2.00	3.00	2.00	3.00
280	4	5	0	3	2	5	7.00	5.67	3.67	5.20	7.00	7.00	7.00	7.00	6.00	6.00	5.00	4.00	4.00	3.00	5.00	5.00	5.00	6.00	5.00
281	4	2	1	1	2	1	3.00	1.33	1.33	2.20	4.00	2.00	3.00	3.00	2.00	1.00	1.00	1.00	2.00	1.00	2.00	2.00	2.00	3.00	2.00
282	5	2	1	3	5	3	6.00	4.33	6.00	3.80	7.00	6.00	5.00	6.00	5.00	3.00	5.00	6.00	6.00	6.00	3.00	4.00	5.00	3.00	4.00
283	1	1	0	4	3	3	2.25	5.00	5.33	3.20	2.00	3.00	2.00	2.00	5.00	5.00	5.00	6.00	5.00	5.00	3.00	2.00	2.00	4.00	5.00
284	2	3	0	1	5	4	6.75	1.67	3.00	5.60	7.00	7.00	6.00	7.00	2.00	2.00	1.00	3.00	3.00	3.00	6.00	5.00	7.00	5.00	5.00
285	1	3	0	1	2	3	6.25	3.33	6.00	5.60	7.00	6.00	7.00	5.00	2.00	5.00	3.00	4.00	7.00	7.00	7.00	5.00	3.00	7.00	6.00
286	5	1	0	1	3	3	1.75	1.67	2.33	1.60	1.00	1.00	3.00	2.00	2.00	1.00	2.00	2.00	3.00	2.00	1.00	2.00	2.00	1.00	2.00
287	1	5	1	4	4	5	6.25	6.67	5.00	6.40	7.00	7.00	6.00	5.00	6.00	7.00	7.00	6.00	6.00	3.00	7.00	7.00	7.00	5.00	6.00
288	3	2	1	2	3	4	1.25	6.00	2.67	5.80	1.00	2.00	1.00	1.00	7.00	6.00	5.00	3.00	2.00	3.00	5.00	6.00	5.00	7.00	6.00
289	3	3	0	3	1	4	6.00	2.00	5.67	5.20	5.00	7.00	6.00	6.00	1.00	4.00	1.00	5.00	6.00	6.00	6.00	5.00	3.00	6.00	6.00
290	3	3	1	3	2	5	3.75	7.00	1.33	5.80	5.00	4.00	3.00	3.00	7.00	7.00	7.00	1.00	1.00	2.00	5.00	7.00	6.00	6.00	5.00
291	3	2	0	4	2	4	6.75	5.67	3.67	4.80	6.00	7.00	7.00	7.00	5.00	6.00	6.00	2.00	4.00	5.00	4.00	4.00	6.00	6.00	4.00
292	2	3	0	1	5	4	5.75	4.33	3.67	3.20	6.00	6.00	5.00	6.00	4.00	4.00	5.00	3.00	3.00	5.00	3.00	2.00	3.00	4.00	4.00
293	3	5	0	3	1	5	6.00	3.67	5.33	4.80	7.00	7.00	5.00	5.00	4.00	5.00	2.00	6.00	5.00	5.00	4.00	6.00	5.00	4.00	5.00
294	5	1	1	1	2	1	1.25	2.67	6.33	5.60	1.00	2.00	1.00	1.00	3.00	2.00	3.00	7.00	7.00	5.00	6.00	5.00	5.00	6.00	6.00
295	3	3	0	1	2	4	5.75	5.00	5.33	4.40	6.00	5.00	6.00	6.00	5.00	5.00	5.00	7.00	5.00	4.00	3.00	5.00	6.00	4.00	4.00
296	3	4	0	4	1	2	3.00	6.33	1.33	5.40	3.00	3.00	2.00	4.00	6.00	6.00	7.00	1.00	2.00	1.00	6.00	5.00	5.00	5.00	6.00
297	3	5	1	3	4	5	4.25	5.33	4.33	4.20	5.00	6.00	3.00	3.00	5.00	5.00	6.00	6.00	5.00	2.00	3.00	4.00	5.00	4.00	5.00
298	3	1	1	4	2	1	6.25	3.67	6.00	5.00	7.00	5.00	7.00	6.00	3.00	5.00	3.00	5.00	7.00	6.00	6.00	5.00	4.00	5.00	5.00
299	2	1	0	2	5	2	5.25	5.67	2.67	4.00	5.00	7.00	4.00	5.00	6.00	6.00	5.00	4.00	2.00	2.00	5.00	4.00	5.00	3.00	3.00

300	2	2	1	3	5	5	3.75	5.67	4.33	4.20	3.00	5.00	3.00	4.00	7.00	5.00	5.00	5.00	6.00	2.00	3.00	5.00	6.00	3.00	4.00
301	5	4	0	5	4	5	3.50	3.67	7.00	4.00	3.00	5.00	4.00	2.00	3.00	6.00	2.00	7.00	7.00	7.00	4.00	5.00	3.00	3.00	5.00
302	5	4	0	3	2	3	3.50	2.33	5.33	2.80	3.00	1.00	5.00	5.00	3.00	2.00	2.00	6.00	6.00	4.00	2.00	3.00	3.00	3.00	3.00
303	3	4	1	4	4	3	6.25	6.67	5.67	6.00	7.00	6.00	7.00	5.00	6.00	7.00	7.00	5.00	5.00	7.00	5.00	7.00	7.00	6.00	5.00
304	4	3	0	3	2	2	4.75	2.00	6.67	3.60	5.00	3.00	6.00	5.00	2.00	2.00	2.00	6.00	7.00	7.00	3.00	5.00	3.00	4.00	3.00
305	4	3	1	5	4	4	6.50	6.67	6.00	6.60	7.00	7.00	7.00	5.00	6.00	7.00	7.00	4.00	7.00	7.00	7.00	5.00	7.00	7.00	7.00
306	3	2	0	3	2	3	5.75	1.67	6.00	2.20	6.00	5.00	6.00	6.00	2.00	1.00	2.00	6.00	6.00	6.00	3.00	1.00	2.00	4.00	1.00
307	5	4	1	1	2	1	1.50	3.67	3.00	2.20	1.00	2.00	2.00	1.00	3.00	6.00	2.00	3.00	3.00	3.00	3.00	1.00	1.00	3.00	3.00
308	5	1	0	2	3	4	1.25	1.33	5.67	5.40	2.00	1.00	1.00	1.00	1.00	2.00	1.00	6.00	5.00	6.00	7.00	5.00	4.00	5.00	6.00
309	4	4	1	2	3	4	5.00	6.33	2.33	5.60	5.00	4.00	6.00	5.00	7.00	6.00	6.00	3.00	2.00	2.00	6.00	6.00	6.00	5.00	5.00
310	1	4	1	3	4	4	6.00	6.67	6.33	5.80	7.00	7.00	5.00	5.00	7.00	7.00	6.00	7.00	7.00	5.00	7.00	6.00	5.00	5.00	6.00
311	4	5	1	5	5	5	6.00	7.00	7.00	6.20	7.00	5.00	5.00	7.00	7.00	7.00	7.00	7.00	7.00	7.00	7.00	7.00	7.00	5.00	5.00
312	3	4	1	4	5	3	6.00	6.00	5.00	5.80	6.00	6.00	6.00	6.00	7.00	5.00	6.00	5.00	7.00	3.00	6.00	6.00	5.00	6.00	6.00
313	3	2	0	4	5	4	2.25	6.33	1.00	4.40	3.00	1.00	2.00	3.00	6.00	6.00	7.00	1.00	1.00	1.00	5.00	3.00	3.00	6.00	5.00
314	4	3	0	3	1	5	6.00	5.67	6.33	3.40	5.00	7.00	5.00	7.00	5.00	6.00	6.00	7.00	6.00	6.00	5.00	3.00	2.00	4.00	3.00
315	4	2	1	4	4	4	5.25	2.67	3.00	3.80	6.00	5.00	5.00	5.00	3.00	2.00	3.00	3.00	3.00	3.00	4.00	3.00	4.00	3.00	5.00
316	1	2	1	5	2	5	6.00	6.67	6.33	6.00	5.00	6.00	6.00	7.00	6.00	7.00	7.00	7.00	7.00	5.00	7.00	5.00	6.00	5.00	7.00
